# The crosstalk between CREB and PER2 mediates the transition between mania- and depression-like behavior

**DOI:** 10.1038/s41386-025-02076-5

**Published:** 2025-02-27

**Authors:** Xin-Ling Wang, Yan-Bin Ji, Su-Xia Li, Tsvetan Serchov

**Affiliations:** 1 Department of Medical Psychology and Ethics, School of Basic Medical Sciences, Shandong University, Ji’nan, 250012 Shandong China; 2 Centre National de La Recherche Scientifque (CNRS), Université de Strasbourg, Institut Des Neurosciences Cellulaires Et Intégratives (INCI) UPR 3212, 67000 Strasbourg, France; 3 Department of Psychiatry and Psychotherapy, Medical Center - University of Freiburg, Faculty of Medicine, University of Freiburg, Hauptstr. 5, 79104 Freiburg, Germany; 4 Department of Neurology, Qilu Hospital of Shandong University, Ji’nan, 250012 Shandong China; 5 National Institute on Drug Dependence, Peking University, Beijing, China; 6 Beijing Key Laboratory of Drug Dependence, Peking University, Beijing, China

**Keywords:** Cellular neuroscience, Bipolar disorder

## Abstract

Bipolar disorder (BD) is a severe psychiatric disorder characterized by alternating manic and depressive episodes. The molecular mechanisms underlying the transition between mania and depression remain unclear. Utilizing a mania animal model induced by ouabain, we observed reduced phosphorylated level of cyclic AMP-responsive element-binding protein (pCREB) and Period (PER)2 expression in the cornu ammonis (CA1) region of the hippocampus, which were restored by lithium treatment. shRNA knockdown of CREB or Per2 in CA1 region induced mania-like behavior, while overexpression of both factors resulted in depression-like behavior. Furthermore, our protein analyses revealed that the upregulation or downregulation of CREB or Per2 influenced each other’s expression. Co-immunoprecipitation results demonstrated that CREB interacts with PER2. Taken together, our data suggest for potential inter-regulatory crosstalk between CREB–PER2 in hippocampal CA1 region, which mediates the transition between mania- and depression-like behaviors.

## Introduction

Bipolar disorder (BD) is a severe psychiatric disorder characterized by alternating manic/hypomanic and depressive episodes. The World Mental Health Survey reported that the lifetime and 12-month prevalence of BD were 2.4% and 1.5%, respectively [1]. Despite its significant impact in the life quality of the patients, pathophysiology of BD is not completely understood. Preventing or modulating the switch between manic and depressive episodes could have significant therapeutic potential for BD. However, due to the limitations of animal models [2, 3], the mechanisms underlying phase transitions in BD remain unclear.

BD is often characterized with dysregulated circadian rhythms, suggesting that clock genes might play critical role in mood regulation and affective states transition in BD [4–16]. This is supported by animal model studies investigating abnormalities in the rhythmic expression of clock genes, as well as clinical research examining the close relationship between clock gene polymorphisms and the occurrence of depression. Lithium, as a first-line treatment of BD, also influences several circadian genes, including *Nr1d1, Gsk3β, Cry1, Arntl, Tim* and *Per2* [17–20]. Per2 is a core component of the circadian clock, linked with the regulation of depression-like behavior [21, 22]. PER2 not only regulates circadian rhythms but also interacts with pathways involved in mood regulation, stress response, and neuroplasticity, suggesting it may serve as a bridge between circadian dysfunction and the neurobiological underpinnings of BD.

Structural and functional abnormalities in several brain regions, including hippocampus, have been observed in individuals with BD, including reduced volume and altered synaptic plasticity [23–25]. One molecular pathway gaining attention in this context involves the cAMP response element-binding protein (CREB), a transcription factor essential for neuroplasticity and the regulation of mood [26, 27]. Indeed, CREB directly influences the expression of *Per2* [28]. Aberrant CREB activity and its downstream effects on Per2 may contribute to the disrupted circadian and mood patterns observed in BD [29], highlighting potential targets for therapeutic interventions.

Experimental models have been instrumental in elucidating the molecular and cellular mechanisms underlying BD. Intracerebroventricular (ICV) administration of the cardiac glycoside and Na^+^/K^+^-ATPase inhibitor ouabain in rats is an commonly used model for studying BD [30, 31], as it initially induces specific manic-like symptoms [3, 31, 32], which later switch to depressive-like behavior. The induced behavioral phenotype of this model can be also reversed by lithium, a key mood-stabilizing medication [30]. Importantly, ouabain has been shown to impact hippocampal function, CREB activity, and circadian gene expression, providing a valuable framework for studying the interplay among these factors in BD pathophysiology [33, 34].

In the present study, using the mania animal model induced by ouabain, we found decreased levels of phosphorylated CREB and PER2 protein expression specifically in the cornu ammonis (CA1) region of the hippocampus. Knockdown of CREB or PER2 by shRNA in this region induced mania-like behaviors, whereas overexpression of those factors led to depression-like behaviors. Protein analysis revealed that changes in CREB or PER2 levels affected the expression of the other. Additionally, co-immunoprecipitation demonstrated an interaction between CREB and PER2. These findings suggest potential inter-regulatory crosstalk between CREB and PER2 in the hippocampal CA1 region, mediating the switch between mania- and depression-like states.

## Methods

### Animals

Sprague Dawley (SD) adult male rats (8–10 weeks old, 220–240 g) were purchased from Charles River Company (No. SCXK–2021–0011, Beijing, China) and housed in groups of three under a 12 h/12 h light/dark cycle (lights on at 8:00, lights off at 20:00) with free access to food and water. The room temperature was maintained at 23 ± 1 °C, with controlled humidity at 50 ± 5%. All experiments were conducted in accordance with the National Institutes of Health Guide for the Care and Use of Laboratory Animals (China) and were approved by the Shandong University School of Basic Medicine Ethics Committee (Ethical approval number: ECSBMSSDU2022-2-51).

### Ouabain-induced mania model

The ouabain-induced mania model was established according to published protocols [30, 35–37]. Ouabain was dissolved in an artificial cerebrospinal fluid (aCSF, 1 mmol/L, 5 μL) and injected intracerebroventricularly (i.c.v.) (coordinates: anterior/posterior, −0.9 mm; medial/lateral, +1.5 mm; dorsal/ventral, −3.3 mm) at a rate of 1 μL/min in rats. The stereotaxic surgery procedure was the same as that used for viral injections.

### Virus construction

For the knockdown of Per2 in the hippocampal CA1 region of rats, we used AAV9-pCAG-EGFP-pU6-shPer2/Scramble (AAV-shPer2/AAV-Scramble), for the overexpression of Per2 in this region, we used LV-pCMV-EGFP-Per2 (LV-Per2) and LV-pCMV-EGFP (LV-control). Additionally, for the KD of CREB in this region, we used AAV9-pCAG-EGFP-pU6-shCREB/Scramble (AAV-shCREB/AAV-Scramble), and for CREB overexpression, we used LV-pCMV-EGFP-CREB (LV-CREB) and LV-pCMV-EGFP (LV-control). The viruses were custom-made from the Shanghai Genechem Co.,Ltd. (China). The vector element maps of the viruses were shown in Supplementary Figs. S2A–S5A. Furthermore, the detailed information of the viruses, and specificity blast results were presented in Supplementary Table S2 and Supplementary Information.

### Stereotaxic surgery and intracerebral microinjection

The protocols are based in previous studies [38]. After anaesthetizing the rats with isoflurane gas (5% concentration), we shaved the fur from the skull and placed them on the stereotaxic apparatus, maintaining anesthesia with isoflurane gas (3% concentration). First, we stereotaxically inserted a cannula guide (internal diameter, 0.45 mm; RWD, Cat# 62001) into the CA1 region (anterior/posterior, −4.3 mm; medial/lateral, ±2.0 mm; dorsal/ventral, −2.8 mm) [39]. Next, we microinjected adeno-associated virus AAV-shPer2/shCREB (1 μL per side) or AAV-Scramble (1 μL per side) bilaterally into the CA1 region using Hamilton syringes (Hamilton, USA, Cat# 80300) connected to injectors (Hamilton, USA, Cat# 62201) via the cannula guide. The injection was performed over 5 min at a rate of 1 μL/min, with the injector left in place for an additional 3 min to allow for diffusion. The lentiviruses (LV-Per2, LV-CREB, and LV-Control) were microinjected in the same manner (1 μL per side). After surgery, the rats were housed individually and received penicillin (20 million units intraperitoneally) once a day for 3 days to prevent infection. Immunofluorescence was performed to validate the viral injection site and expression.

### Drug administration

Drug administration was performed as previously described [35]. After ouabain microinjection surgery, rats received either 0.9% saline (vehicle) or lithium carbonate (20 mg/kg, 2 mL/kg) intraperitoneally once daily at 9:00 a.m. (ZT 01) for 7 consecutive days. Lithium carbonate was dissolved in saline at a concentration of 2 mg/ml.

We conducted the sucrose preference test, forced swimming test, open field test, and elevated plus maze test to assess mania- and depression-like behaviors, following methods from previous studies [30, 31]. The behavioral test procedures described below were based on earlier studies conducted in our lab [40].

### Sucrose preference test (SPT)

Rats were singly housed and habituated to water or a 1% sucrose solution for 48 h before the SPT. The positions of the bottles were interchanged in the middle of the habituation phase. The SPT was performed after the rats were deprived of food and water for 4 h. The rats were given two bottles: one containing tap water (W) and the other 1% sucrose solution (S). The positions of the two bottles were interchanged half an hour later to avoid side bias. The consumption of the sucrose solution and tap water over 1 h was quantified by measuring the weight of each bottle. Sucrose preference was calculated as [Weight S/ (Weight S + Weight W)] ×100.

### Forced swim test (FST)

Rats were placed in a glass barrel (25 cm in diameter) filled with water (45 cm in depth) at 23 ± 1 °C under bright light conditions and video recorded for 5 min. Cumulative immobility time was measured with a stopwatch. Immobility was defined as no movement of the body, tail and limbs, while mobility was recognized as swimming or struggling behaviors. Prolonged immobility time was considered indicative of despair, whereas shortened immobility time was identified as mania-like behavior.

### Open field test (OFT)

Rats were placed in a Plexiglas open-field arena (42 cm × 42 cm × 42 cm), and locomotion was monitored and tracked using an automated system (SMART v2.5.21, Panlab, USA) for 5 min. The arena was divided into 25 equally sized squares with the central 9 squares defined as the central zone and the remaining squares as the corners. The cumulative time spent in the central zone and the total distance traveled were recorded.

### Elevated plus maze (EPM) test

The elevated plus maze consists of a central platform with two closed arms (50 cm in length, 10 cm in width, 40 cm in height, with walls on both sides) and two open arms of the same size without walls. The maze was positioned 100 cm above the floor. A rat was placed in the center of the platform, facing an open arm, and allowed to explore the maze freely for 5 min. Time spent in the open arms and the number of entries into the open arms were analyzed using SMART v2.5.21 software (Panlab, USA). The platform was cleaned with 75% ethanol after each test.

### Tail suspension test (TST)

This test was performed according to our previous study [22]. Rats were attached with their tails (1–1.5 cm from the tip of the tail) to a horizontal bar located. This was performed for 6 min and the immobility time was used in the analysis. Rats who climb their tails (>10% of total time) were excluded for analysis.

### Brain tissue collection

For quantitative molecular analysis, experiments were conducted in parallel with the behavioral studies, the ouabain injected rats were decapitated at ZT02 and ZT14, while the rest of the animals were killed only at ZT02. The brains were collected and frozen at −80 °C for subsequent western blot analysis. The mPFC, NAc, CA1, CA3 and DG hippocampal regions (including pulled dorsal and ventral parts) were then isolated by microdissection using a cryostat microtome.

### Western blotting

Tissues were homogenized in a RIPA lysis buffer (Macklin, cat#R917927-100 ml) mixed with protease inhibitor cocktail (Sigma-Aldrich, cat#P8849-1ML) and phosphatase inhibitor mixture (Innochem, cat#B2248). The samples were then centrifuged at 10,000 × *g* for 30 min at 4 °C, and the supernatant was collected and quantified using the Microplate BCA Protein Assay Kit (Thermo Fisher Scientific). Equal amounts of protein were separated on 10% SDS-PAGE gels, and the proteins were then transferred onto a hydrophobic PVDF transfer membrane (Millipore, cat# SJHVM4710). The membranes were washed with TBST (Tris-buffered saline with 0.1% Tween 20), blocked with 5% BSA for 1 h, and incubated with primary antibodies overnight at 4 °C. The following primary antibodies were used: mouse anti-GAPDH (1:5000, Bioss), mouse anti-Per2 (1:1000, Proteintech), rabbit anti-phospho-CREB (Ser133) (1:1000, Cell Signaling Technology), anti-PER1 (1:1000, Proteintech, cat#13463-1-AP) and rabbit anti-CREB (1:1000, Proteintech). The next day, after three 5-minute washes with TBST, the membranes were incubated with HRP-conjugated secondary antibodies (1:2,000 concentration, Goat Anti-mouse IgG Antibody, Bioss, cat#bs-0296G-HRP, or Goat Anti-rabbit IgG Antibody, Bioss, cat#bs-0295G-HRP, used according to the host species of the primary antibodies) for 1 h at room temperature, followed by three additional 5-minute washes with TBST. Protein bands were visualized using a Western chemiluminescent HRP substrate (Millipore, cat# WBKLS0100), and the bands were imaged using a fluorescent imager (Tanon-5200). Protein levels were normalized to GAPDH on the same gel, and all quantifications were performed using Image J software.

### Immunohistochemistry

Rats that underwent behavioral tests were anesthetized, and intracardiac perfusion was performed with phosphate-buffered saline (PBS), followed by 4% paraformaldehyde (PFA). The brains were postfixed in PFA for 24 h at 4 °C and then transferred to 30% sucrose in PBS. Coronal sections were cut using a freezing microtome (Leica, CM1950) at a thickness of 25 μm. After three rinses in PBS, 5 min each, the brain slices were mounted on glass slides with antifade mounting medium (Solarbio). Fluorescent EGFP images were captured using a full-field digital slice scanning microscope (VS120, Olympus, Japan) and analyzed using OlyVIA software (Olympus, Japan).

### Co-immunoprecipitation assay

The co-immunoprecipitation assay was performed to test the interaction between PER1, PER2, and CREB proteins in cells from the hippocampal CA1 region of normal adult rats. Total protein lysates were obtained using an immunoprecipitation buffer (50 mM Tris–HCl, pH 8.0, 150 mM NaCl, 5 mM EDTA, 0.5% NP-40, 2 μg/ml aprotinin, 1 μg/ml leupeptin, 1 mM PMSF, 1 mM sodium vanadate and 10 mM sodium fluoride). The lysates were pre-cleared using protein A/G-agarose beads, and total protein in supernatants was qualified using the BCA method. Next, 1 μg/μL of total protein in PBS was mixed with primary antibodies against PER1 (Ca# 13463-1-AP, Proteintech, Concentration 1:1000), PER2 (Ca# 67513-1-Ig, Proteintech, Concentration 1:000), CREB (Ca# 12208-1-AP, Proteintech, Concentration 1:1000), or IgG as a control. The mixtures were shaken on a rotating shaker at 4 °C overnight. The supernatants were then collected for immunoblotting. For immunoblotting, 30 μg of protein samples were separated by SDS-PAGE, and after transfer to a PVDF membrane, the blots were probed overnight with the respective primary antibodies (PER1 (Ca# 13463-1-AP, Proteintech, Concentration 1:1000), PER2 (Ca# 67513-1-Ig, Proteintech, Concentration 1:000), CREB (Ca# 12208-1-AP, Proteintech, Concentration 1:1000)). After three washes with TBST, the blots were incubated with HRP-conjugated secondary antibodies. Signals were visualized using the Excellent Chemiluminescent Substrate Detection Kit (Thermo, NCI5079) [41]. β-actin was used as the internal control.

### Quantification and statistical analysis

All data were analyzed using GraphPad Prism 8.0.0 software and are presented as mean ± SEM. Behavioral and western blot data were statistically analyzed using two-way analysis of variance (ANOVA) followed by Tukey’s post-hoc test to compare the means of two- factors, two-tailed unpaired Student’s *t* test or Mann–Whitney test to compare the means of two groups. *P* ≤ 0.05 was considered statistically significant (*P ≤ 0.05, **P ≤ 0.01, ***P ≤ 0.001). Prior to statistical analyses data assumptions (normality and homoscedasticity of the distributions) were assessed by Shapiro–Wilk and Kolmogorov–Smirnov tests by GraphPad Prism 8.0.0 software. Detailed statistical tests, *P* values, sample size, related parameters, exclusions of data/samples and their corresponding reasons are provided in Supplementary Table S1. Statistical analyses are briefly mentioned in the figure legends. For all molecular and behavioral studies, rats were randomly assigned to groups. Additionally, experimenters were blinded to the group assignments until data collection was completed. Sample sizes were determined based on extensive laboratory experience.

## Results

### pCREB and PER2 levels in the CA1 region of the hippocampus are decreased in the ouabain-induced mania model

**Fig. 1:**
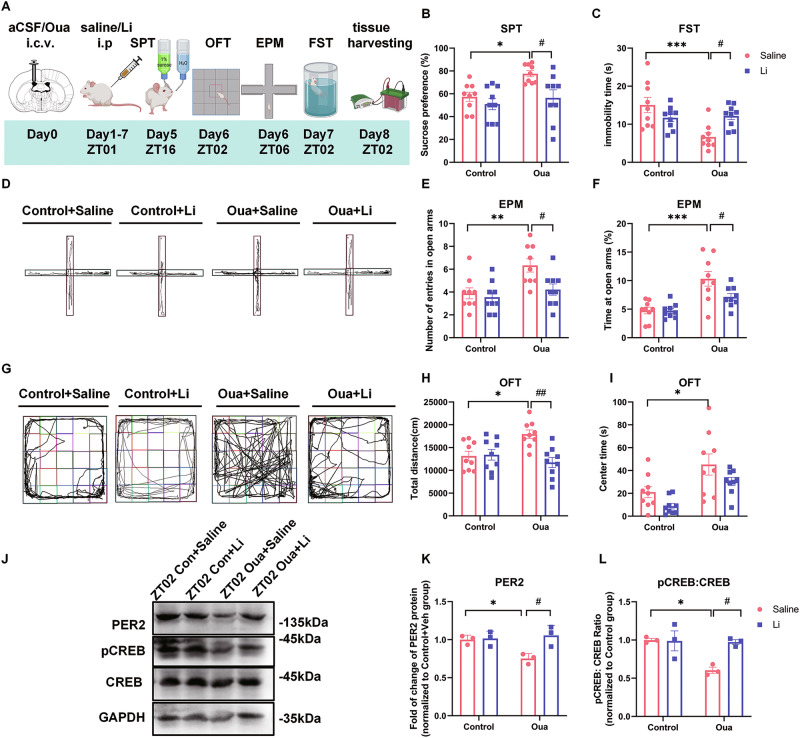
pCREB and PER2 levels in the CA1 region of the hippocampus are decreased in the ouabain-induced mania model. **A** Experimental design of the ouabain model and behavioral analyses: the rats are i.c.v. infused (day0) with aCSF or ouabain (Oua), daily i.p. injected (day1–7) with saline or lithium carbonate (Li), behaviorally tested (day5–7) and sacrificed for brain tissue harvesting. The effects of aCSF (control)/Oua infusion and the following saline/Li treatment (*n* = 9 rats per group) on sucrose preference (**B**) in sucrose preference test (SPT) and immobility time (**C**) in forced swim test (FST). **D** Representative images of rat traces in the elevated plus maze test (EPM). Effects of Oua and Li on the number of entries (**E**) and time spent (**F**) in the open arms of EPM test. **G** Representative images of rat traces in the in the open field test (OFT). Effects of Oua and Li on total distance traveled (**H**) and time spent in the central zones (**I**) of the OFT. **J** Representative western blots showing PER2, pCREB and CREB protein expression in CA1 area at ZT02. Quantification of relative PER2 protein levels normalized to GAPDH (**K**) and pCREB:CREB ratio (**L**) (*n* = 3 rats per group). Two-way ANOVA with Tukey’s post-hoc test: *P < 0.05, **P < 0.01, ***P < 0.001 vs. control + vehicle group; #*P* < 0.05, ##*P* < 0.01 vs. ouabain + vehicle group). Data are presented as mean ± SEM and the individual data points are depicted. See also Supplementary Fig. S1 and Supplementary Table S1. Some of the sketches were made with biorender.com.

In order to investigate the mechanism of affective states transition in BD and the role of PER and CREB, we used the ouabain-induced model of BD. In this paradigm ouabain dissolved in artificial cerebrospinal fluid (aCSF) (used as the control) is infused intracerebroventricularly (i.c.v.) in rats (1 mmol/L, 5 μl), as described in previous reports [30, 31, 42]. As mood stabilizing drug, we administered intraperitoneally lithium carbonate (20 mg/kg), dissolved in saline (vehicle), once per day for 7 consecutive days [36, 43–45]. The rats were behaviorally characterized by the battery of tests, including sucrose preference test (SPT), forced swim test (FST), open field test (OFT), and elevated plus maze (EPM) test to assess anhedonia, depression-like, impulsivity-like behaviors, as well as locomotor and exploratory activity (Fig. 1A) [30, 31, 42]. Five days after ouabain administration, sucrose preference was significantly increased in the ouabain group compared to the controls, and this effect was significantly ameliorated by lithium carbonate treatment (Fig. 1B). On day 7, the immobility time in FST was significantly reduced in the ouabain group compared to the control group, which was also significantly ameliorated by lithium (Fig. 1C). In the EPM test, the number of entries and time spent in the open arms were significantly increased by ouabain, both of which were reversed by lithium treatment (Fig. 1D–F). In the OFT, the total distance traveled in the ouabain group was significantly greater than in the control group (Fig. 1G, H). Additionally, the time spent in the central zones was significantly increased in the ouabain group compared to the control group (Fig. 1G, I). Thus, as expected, ouabain resulted in mania-like behavior 1 week after the administration, characterized by increased sucrose preference, locomotor activity and impulsivity-like behavior, as well as decreased depression-like behavior, all of which were reversed by lithium treatment. In order to further confirm the face validity of the ouabain model and assess potential affective state transition to depression-like behavior, the rats were subjected to tail suspension test (TST) 2 weeks after the ouabain application. Indeed, our results show significantly increased immobility time in TST (Supplementary Fig. S1K).

To explore the molecular changes underlying these behavioral effects, we conducted a western blot analysis on proteins extracted from several brain regions, including mPFC, NAc, as well as hippocampal CA1, CA3 and DG areas at ZT02 and ZT14 (corresponding to the peak and minimum points of PER2 protein expression) [46] one week after the aCSF/ouabain injection (Fig. 1A, Supplementary Fig. S1A). We found that PER2 levels in CA1 region at ZT02 and in CA3 region at ZT14 were significantly decreased in the ouabain group, compared to the control group (Supplementary Fig. S1C, D). In contrast, in the other analyzed brain regions, including mPFC, NAc and DG, there were any significant difference in the PER2 levels between the two groups at ZT02 or ZT14 (Supplementary Fig. S1E–G). Moreover, our results show that pCREB levels in the CA1 region, alongside PER2, were significantly decreased in the ouabain group compared to the control group, and these effects were significantly ameliorated by lithium (Fig. 1J–L). In contrast, we did not find any significant differences in total CREB expression between the groups (Supplementary Fig. S1R). We also explored the molecular changes at ZT2 and ZT14 by western blot two weeks after the ouabain injection, when we observed transition to depression-like behavior (Supplementary Fig. S1K). However, we didn’t find any significant differences in the PER2, pCREB, PER1 and CREB levels among the groups (Supplementary Fig. S1L–Q).

### Knockdown (KD) of *Per2* in the CA1 region produces mania-like behavior and induces downregulation of CREB levels

**Fig. 2:**
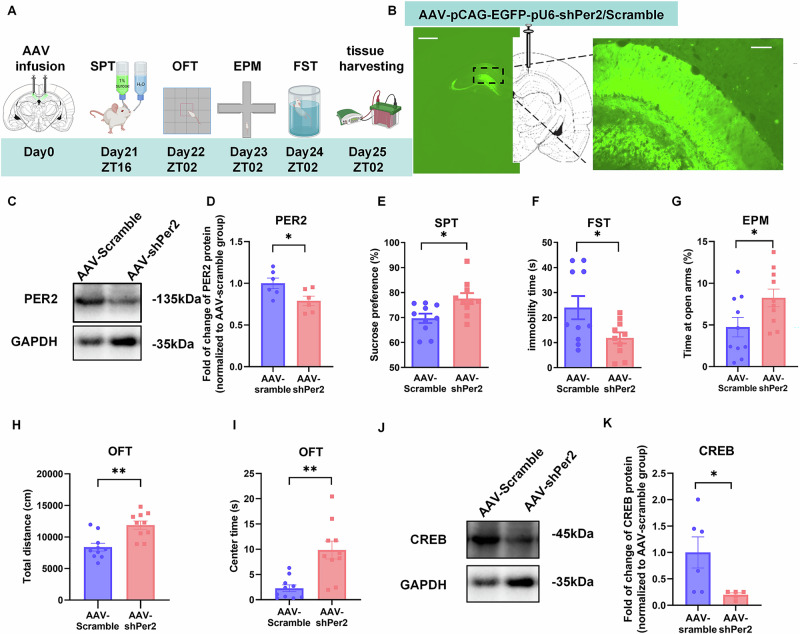
Knockdown of *Per2* in the CA1 region induces mania-like behaviors and downregulates CREB levels. **A** Timeline of the AAV microinjections into CA1 and behavioral tests. **B** Injection site of AAV-pCAG-EGFP-pU6-shPer2/Scramble (left panel, scale bar: 1 mm) and viral EGFP expression (right panel, scale bar: 100 µm) in the CA1 region. Representative western blot (**C**) and quantification of PER2 protein levels (**D**) (*n* = 6 rats per group). The effects of AAV-shPer2/scramble infusion into the CA1 region (*n* = 10 rats per group) on sucrose preference in SPT (**E**), immobility time in the FST (**F**), time spent in open arms of EPM (**G**), total distance (**H**) and time spent in central zone (**I**) of OFT. Representative western blot (**J**) and quantification of the relative protein levels of CREB in CA1 at ZT02 (**K**) (*n* = 5–6 rats per group). **D**–**H**: Two-tailed Student’s *t* test, **I** & **K**: Mann–Whitney test: *P < 0.05, **P < 0.01). Data are presented as mean ± SEM and the individual data points are depicted. See also Supplementary Fig. S2 and Supplementary Table S1. Some of the sketches were made with biorender.com.

To investigate the role of *Per2* expression in mood regulation, we knocked down *Per2* in CA1 region and conducted behavioral tests (Fig. 2A). Rats were microinjected with AAV-pCAG-EGFP-pU6-shPer2/Scramble (AAV-shPer2/AAV-scramble) into the CA1 region (Fig. 2B, Supplementary Fig. S2A). 21 days later, we validated the injection/knockdown site of the CA1 region by immunofluorescence (Fig. 2B), and our western blot analysis confirmed that PER2 levels were significantly downregulated by AAV-sh*Per2* (Fig. 2C, D). Our behavioral analyses show significantly increased sucrose preference values in the AAV-sh*Per2* group compared to the control (AAV-Scramble) group (Fig. 2E). Additionally, immobility time in the FST was significantly reduced in the *Per2*KD group compared to the control group (Fig. 2F), indicating antidepressant-like behaviors. In the EPM test, the time spent in the open arms was significantly increased in the AAV-sh*Per2* group compared to the control group (Fig. 2G, Supplementary Fig. S2B). However, there was no significant difference between the two groups in the number of entries into the open arms (Supplementary Fig. S2B, C). In the OFT, the total distance traveled was significantly greater in the AAV-sh*Per2* group compared to the control group (Fig. 2H, Supplementary Fig. S2D), indicating hyperactivity. Moreover, the time spent in central zones was also significantly increased in the AAV-sh*Per2* group (Fig. 2I, Supplementary Fig. S2D), demonstrating increased exploratory and impulsivity-like behavior. Furthermore, CREB levels in the CA1 region were significantly reduced in the AAV-sh*Per2* group, compared to the AAV-Scramble group (Fig. 2J, K), indicating that knockdown of *Per2* in CA1 downregulates CREB levels in this region. However, there were not any significant differences of the pCREB or PER1 levels in CA1 induced by AAV-sh*Per2* (Supplementary Fig. S2E–G).

Therefore, knockdown of *Per2* in the CA1 leads to mania-like behavior—evidenced by increased sucrose preference and locomotor activity, along with decreased impulsivity- and depression-like behavior. Additionally, it downregulates CREB levels in this region.

### Overexpression of *Per2* in CA1 region induces depression-like behavior, ameliorates ouabain-induced mania-like behaviors and upregulates CREB levels in this region

**Fig. 3:**
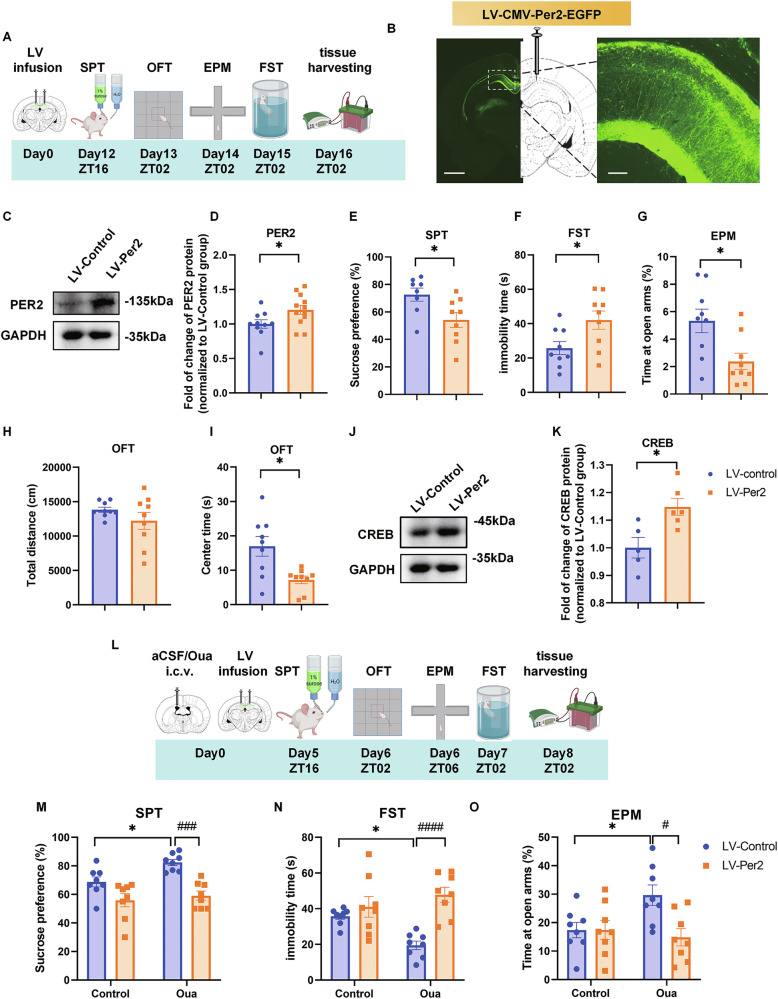
Overexpression of *Per2* in the CA1 region induces depression-like behaviors, upregulates CREB levels and rescues ouabain-induced mania-like behaviors. **A** Timeline of the LV microinjections into CA1 and the behavioral experiments. **B** Injection site of LV-CMV-Per2-EGFP (left panel, scale bar: 1 mm) and viral EGFP expression (right panel, scale bar: 100 µm) in the CA1 region of the rat brain. Representative western blot (**C**) quantification of PER2 protein levels (**D**). Effects of Per2 overexpression in the CA1 region (*n* = 9 rats per group) on sucrose preference in the SPT (**E**), immobility time in the FST (**F**), time spent in the open arms of EPM (**G**), total distance (**H**) and time spent in the central zone of OFT (**I**). Representative western blots (**J**) and quantification of the relative protein levels of CREB in CA1 at ZT02 (**K**) (*n* = 5–6 rats per group). **D**–**G**, **K**: Two-tailed Student’s *t* test, **H**, **I**: Mann–Whitney test: *P < 0.05. **L** Experimental timeline: rats are i.c.v. infused with aCSF/ouabain, simultaneously microinjected with LV-CMV-Per2-EGFP in CA1 region and behaviorally tested after day12. Effects of CA1 Per2 overexpression in the ouabain model on sucrose preference (**M**), immobility time in FST (**N**) and time spent in the open arms of EPM (**O**). (*n* = 8 rats per group, two-way ANOVA with Tukey’s post-hoc test: *P < 0.05, #*P* < 0.05, ####*P* < 0.0001). Data are presented as mean ± SEM and the individual data points are depicted. See also Supplementary Fig. S3 and Supplementary Table S1. Some of the sketches were made with biorender.com.

To further examine the function of PER2 in the CA1 region in mood modulation, we overexpressed *Per2* in the CA1 via microinjection of lentivirus LV-pCMV-EGFP-Per2 (LV-*Per2*) or LV-pCMV-EGFP (LV-control) (Supplementary Fig. S3A, Supplementary Table S2) into this region and performed behavioral tests (Fig. 3A). The immunofluorescence histology experiments confirmed the infusion site in the CA1 region (Fig. 3B), while western blot analysis revealed that the PER2 levels were significantly upregulated in the CA1 region by LV-*Per2* 12 days after the injections (Fig. 3C, D). The behavioral analyses show that sucrose preference values were significantly reduced in the LV-*Per2* group compared to the control group (Fig. 3E). In the FST, the immobility time was significantly prolonged in the LV-*Per2* group (Fig. 3F). Furthermore, in both the EPM and OFT, the time spent in the open arms and central zones was significantly reduced in the LV-*Per2* group compared to the control group (Fig. 3G–I; Supplementary Fig. S3B, D). Additionally, western blot analysis of CA1 proteins showed that CREB levels were significantly elevated in the LV-*Per2* group (Fig. 3J, K), but the pCREB and PER1 levels were not significantly altered (Supplementary Fig. S1E–G). Therefore, overexpression of *Per2* in CA1 region induced depression- and impulsivity-like behaviors and increased CREB levels in this region.

To further confirm the role of *Per2* in the pathogenesis of BD, we infused ouabain into the ICV to model BD and simultaneously microinjected LV-*Per2* into the CA1 to examine whether it could reverse the ouabain-induced mania-like behavior. Behavioral tests, including the SPT, OFT, EPM, and FST, were performed from the 5th to 7th day after injection of aCSF/ouabain and lentivirus (Fig. 3L). In the SPT, the Per2 overexpression significantly downregulated the ouabain enhanced sucrose preference values (Fig. 3M). Likewise, the immobility time in FST that was decreased in the ouabain + LV-control group, was significantly attenuated by LV-*Per2* (Fig. 3N). In the EPM, time spent in the open arms was significantly increased by ouabain and significantly reduced by LV-*Per2*, without affecting the the number of entries into the open arms (Fig. 3O; Supplementary Fig. S3H, I). In the OFT, both total distance traveled and time spent in the central zones were significantly increased by ouabain infusion, and these effects were significantly moderated by LV-*Per2* (Supplementary Fig. S3J–L). Thus, LV-*Per2* exhibits an antimanic-like effect, including the amelioration of immobility time in the FST, time spent in the open arms in EPM, as well as total distance traveled and time spent in the central zones in the OFT, all induced by ouabain.

### Knockdown of *CREB* in CA1 induces mania-like behaviors and decreases PER2 levels

**Fig. 4:**
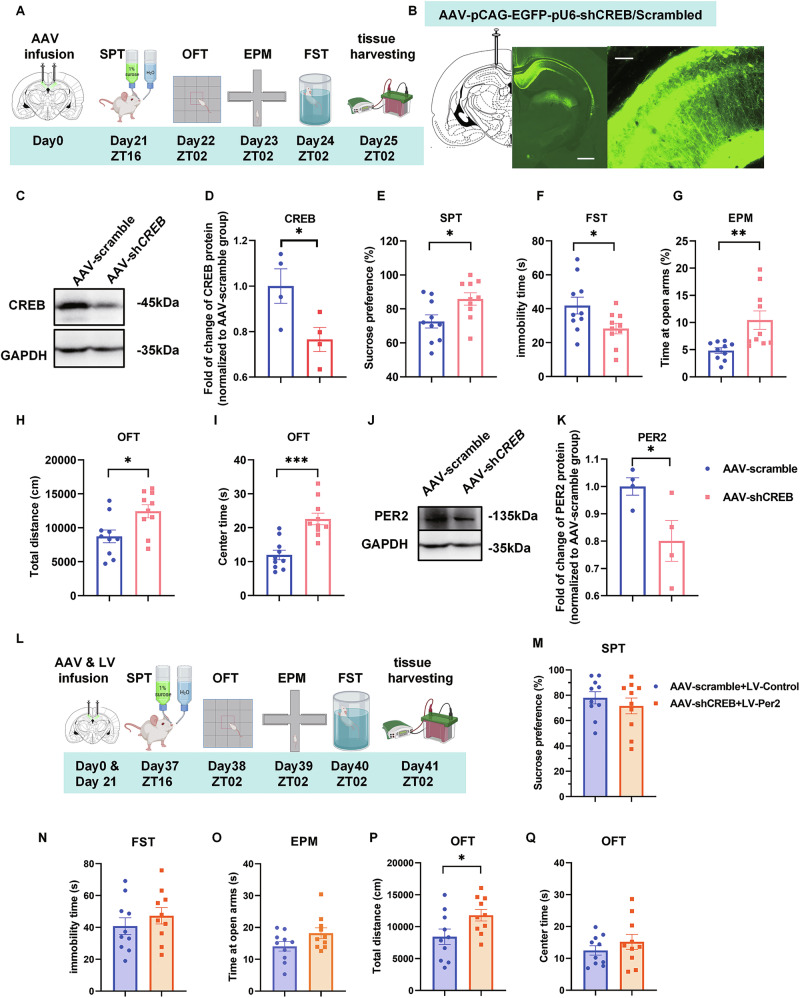
Knockdown of *CREB* in the CA1 region induces mania-like behaviors and decreases PER2 levels, which can be partially moderated by the overexpression of *Per2* in CA1. **A** Timeline of the AAV microinjections and the behavioral experiments. **B** Injection site of AAV-pCAG-EGFP-pU6-shCREB/Scramble (left panel, scale bar: 1 mm) and viral EGFP expression (right panel, scale bar: 100 µm) in the CA1 region. Representative western blots (**C**) and quantification of the relative protein levels of CREB in CA1 at ZT02 (**D**) (*n* = 4 rats per group). Effects of CREB knockdown in the CA1 region (*n* = 10 rats per group) on sucrose preference in SPT (**E**), immobility time in FST (**F**), time spent in the open arms of EPM (**G**), total distance (**H**) and time spent in the central zone of OFT (**I**). Representative western blots (**J**) and quantification of the relative protein levels of PER2 (**K**) (*n* = 4 rats per group). **L** Experimental timeline. Sucrose preference (**M**), immobility time in FST (**N**), time spent in the open arms of EPM (**O**), total distance (**P**) and time spent in the central zone of OFT (**Q**) (*n* = 10 rats per group). **D**–**F**, **H**–**Q**: Two-tailed Student’s *t* test, **G**: Mann–Whitney test: *P < 0.05, **P < 0.01, ***P < 0.001). Data are presented as mean ± SEM and the individual data points are depicted. See also Supplementary Fig. S4 and Supplementary Table S1. Some of the sketches were made with biorender.com.

To explore the role of CREB, one of the transcriptional regulators of *Per2*, in mood-related behaviors and its interaction with *Per2*, we knocked down *CREB* by infusing AAV-pCAG-EGFP-pU6-shCREB (AAV-shCREB) or AAV-pCAG-EGFP-pU6-Scramble (AAV-scramble) (Supplementary Fig. S4A, Supplementary Table S2) bilaterally into the CA1. Behavioral tests were conducted 21 days later (Fig. 4A). Immunofluorescence validated the infusion site (Fig. 4B), and western blot analysis confirmed that CREB protein levels were significantly downregulated by AAV-shCREB (Fig. 4C, D). Notably, sucrose preference values were significantly increased, and the immobility time in FST was significantly reduced in the AAV-shCREB group compared to the control group (Fig. 4E, F). In the EPM test, both the number of entries into the open arms and the time spent in the open arms were significantly increased in the *CREB* KD group compared to the control group (Fig. 4G, Supplementary Fig. S4B, C). Furthermore, both total distance traveled and time spent in the central zones of the OFT were significantly increased in the *CREB* KD group compared to the control group (Fig. 4H, I; Supplementary Fig. S4D). Thus, *Creb* knockdown in the CA1 induced mania-like behavior, enhanced exploratory behavior and hyperactivity. In a parallel experiment, western blot analysis showed that PER2 levels were also significantly decreased by AAV-shCREB (Fig. 4J, K), demonstrating that KD of *CREB* downregulates PER2 levels. Besides, the pCREB levels were also significantly reduced by AAV-shCREB, while, the PER1 levels were not significantly affected (Supplementary Fig. S4H–J).

Next, we infused LV-*Per2* bilaterally into the CA1 to examine whether *CREB* KD-induced behaviors could be ameliorated by *Per2* overexpression. 12 days post lentivirus injection, behavioral tests were also performed between the two groups (Fig. 4L). Sucrose preference values and immobility time showed no significant differences in the AAV-shCREB + LV-*Per2* group compared to the AAV-scramble + LV-control group (Fig. 4M, N). In the EPM test, the time spent in the open arms and the number of entries into the open arms were not significantly different between the control and AAV-shCREB + LV-*Per2* groups (Fig. 4O; Fig. S4F, G). In the OFT, the time spent in the central zones also showed no significantly differences between the AAV-shCREB + LV-*Per2* group and the AAV-scramble + LV-control group (Fig. 4Q); however, the total distance traveled in this group showed the same trend as before the injection of LV-*Per2* (Fig. 4P; Supplementary Fig. S4E). Thus, *Per2* overexpression in the CA1 ameliorated the behaviors induced by *CREB* knockdown.

### Overexpression of *CREB* in the CA1 causes depression-like behaviors and induces upregulation of PER2 levels

**Fig. 5:**
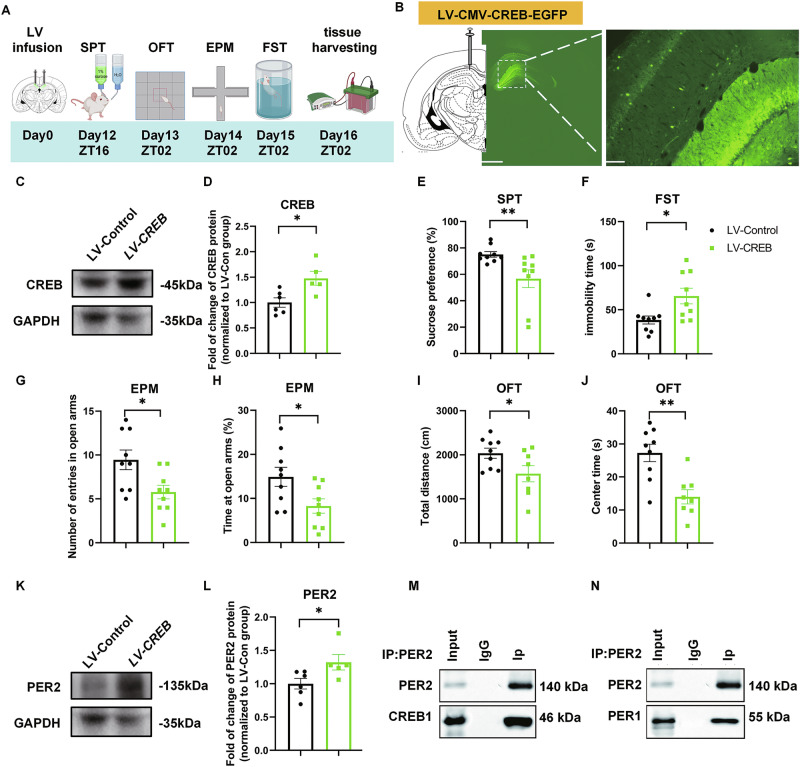
Overexpression of *CREB* in the CA1 region induces depression-like behaviors and increases PER2 levels. **A** Timeline of the LV microinjections and the behavioral experiments. **B** Injection site of LV-CMV-CREB-EGFP (left panel, scale bar: 1 mm) and viral EGFP expression (right panel, scale bar: 100 µm) in the CA1 region of the rat brain. Representative western blots (**C**) and quantification of the relative protein levels of CREB in the CA1 at ZT02 (**D**) (*n* = 5 rats per group). The effects of CREB overexpression in the CA1 region (*n* = 9 rats per group) on sucrose preference (**E**), immobility time in FST (**F**), the number of entries (**G**) and time spent (**H**) in the open arms of EPM, total distance (**I**) and time spent in the central zone (**J**) of OFT. Representative western blots (**K**) and quantification of the relative protein levels of PER2 in the CA1 region at ZT02 (**L**) (*n* = 5–6 rats per group). **D**, **F**–**L**: Two-tailed Student’s *t* test, **E**: Mann–Whitney test: *P < 0.05, **P < 0.01). **M** Co-immunoprecipitation experiment conducted with lysates from rat hippocampal CA1 region showing that CREB1 and PER2 co-immunoprecipitate. **N** Co-immunoprecipitation experiment showing that PER1 and PER2 can also be co-immunoprecipitated with CREB1. Data are presented as mean ± SEM and the individual data points are depicted. See also Supplementary Fig. S5 and Supplementary Table S1. Some of the sketches were made with biorender.com.

To further investigate the role of CREB and its potential crosstalk with PER2 in mood-related behaviors, we overexpressed *CREB* by infusing LV-pCMV-EGFP-pUbc-CREB1(LV-Creb1) or LV-pCMV-EGFP (LV-control) (Supplementary Fig. S5A, Supplementary Table S2) bilaterally into the CA1 (Fig. 5B) and conducted behavioral tests 12 days later (Fig. 5A). Western blot analysis confirmed that CREB levels were significantly increased by LV-CREB (Fig. 5C, D). Sucrose preference values were significantly decreased, and immobility time was significantly increased in the LV-CREB group (Fig. 5E, F). In the EPM test, both the number of entries and the time spent in the open arms were significantly decreased in the LV-CREB group (Fig. 5G, H; Fig. S5B). In the OFT, total distance traveled and time spent in central zones were both significantly reduced by LV-CREB (Fig. 5I, J, Supplementary Fig. S5C). In a parallel experiment, PER2 and PER1 levels were also significantly upregulated by LV-CREB (Fig. 5K, L; Fig. S5D, F), while the pCREB levels were not significantly altered (Supplementary Fig. S5D, E).

Thus, overexpression of *CREB* in the CA1 results in depressive- and impulsivity-like behaviors and upregulates PER2 levels in this region.

### The interaction between CREB and PER2

To explore the potential direct interaction between CREB and PER2, we conducted co-immunoprecipitation (Co-IP) experiments with proteins extracted at ZT02 from the CA1 region of rats. Our results show that PER2 and CREB were co-immunoprecipitated (Fig. 5M). Additionally, PER1 and PER2 were also co-immunoprecipitated (Fig. 5N). These findings suggest that CREB and PER2 may physically interact.

## Discussion

The molecular mechanisms underlying the transition between mania and depression in BD remain unclear. Here, we found that ouabain-induced mania-like behaviors were accompanied by downregulation of pCREB/CREB and PER2 levels in the hippocampal CA1 region. Furthermore, the antimanic agent lithium upregulated pCREB/CREB and PER2 levels in CA1. KD of *CREB* and/or *Per2* using RNAi resulted in mania-like behaviors, while their overexpression in this region led to depression-like behaviors. Additionally, overexpression or KD of *CREB* respectively increased or downregulated PER2, and vice versa. Co-IP experiments showed that PER2 and CREB co-immunoprecipitated. These findings suggest that CREB–PER2 crosstalk may represent a potential molecular mechanism underlying the transition between manic and depressive episodes in bipolar disorder (Supplementary Fig. S6).

Initially, we showed that ouabain, a Na+K+ATPase inhibitor, successfully induced mania-like behavior, which could be attenuated by lithium, consistent with previous studies [42, 47]. Our data here indicates that alternation of pCREB and PER2 may be involved in this process, supporting multiple studies suggesting that CREB/PER2 play a role in the treatment of BD [48–52]. We have explored several mood-related brain regions, including mPFC, NAc and hippocampal CA1, CA3 and DG areas, at 2 different ZT points, and we found specific downregulation of PER2 protein and pCREB levels in CA1 region at ZT02. However, we do not exclude that ouabain might have also phase shifting effects on the circadian clock gene expression, as reported previously in cultured chick pineal cells [53], that cannot be observed here due to the insufficient time points data. Hence, we have limited our analysis on CA1 area and ZT02 for our further experiments and didn’t investigate the circadian function of PER2, which is a limitation of this study.

Though, the ouabain model also exhibited depressive-like phenotype two weeks after ouabain administration, which is in line with previous studies [35], we didn’t find any significant difference in the PER2 or pCREB/CREB levels in the CA1 region, indicating potential differences in the transition mechanism in this model. In the future, the mechanism underlying the ouabain model and its mood transition needs further investigation.

cAMP response element-binding protein (CREB) plays a key role in the pathogenesis of mood disorders. Gaspar et al. showed that elevated levels of pCREB/CREB are found in primary fibroblasts from patients in the bipolar depression state [54], consistent with our results in rats. As a convergence point for various cellular signals, CREB is phosphorylated at Ser133 [29, 55–57]. The pCREB is a known transcriptional regulator of numerous genes containing CREB binding sites [29, 57], including the circadian clock genes *Per1* and *Per2*. Overexpression of *CREB* in hippocampal CA1 induces depression-like behaviors, while KD of *CREB* in this region leads to mania-like behavior, consistent with previous reports [26, 29, 58, 59]. We found that CREB can positively regulate PER2 levels, and vice versa. Consistent with our findings, McCarthy et al. also demonstrated that KD of *CREB* in fibroblasts from BD patients leads to a reduction in the baseline amplitude of PER2 rhythmicity [15]. The AAV and LV vectors used in this study lacked cell-type specificity, as the complementary effects of different cell types may influence clock gene expression. Indeed, it has been reported that the localization of CREB in the brain is cell-type specific: it is limited to certain types of glial cells and astrocytes [60]. Therefore, CREB may exert its effects in glial cells. Furthermore, in the CREB–PER2 crosstalk, PER2 may be the core protein mediating mood transitions, as it is known to play various roles not only in circadian rhythms, but also in the cardiovascular system, cell cycle, metabolism, nervous system, and immune system [61].

Many studies indicate the involvement of *Per1* and *Per2* in patients with depression and bipolar disorder [19, 62], with increasing reports highlighting the role of *Per2* in BD [35, 50–52, 63–65]. In our previous study, we demonstrated that KD of *Per1* in the hippocampal CA1 region resulted in enhanced depression-like behaviors, such as a reduction in sucrose preference and prolonged immobility time in the FST, while knockdown of *Per2* produced the opposite effect in the FST (Wang et al., BioRxiv, 2021) [66]. We interpreted this as antidepressant-like or mania-like behavior. In studies of BD treatment, Moreira et al. identified several clock genes associated with the response to lithium, including *Per* [18]. Li et al. also demonstrated that lithium increased *Per2* expression at both the transcriptional and posttranscriptional levels [20]. Furthermore, Kim and colleagues found that lithium upregulated *Per2* and early growth response protein 1 (ERG1) [50]. A recent study by Zhou et al. showed that lithium increased *Per2* expression by decreasing the expression of the transcription factor E4 promoter-binding protein 4 (E4BP4) [52]. In line with their findings, we observed that lithium rescued the ouabain-induced mania-like behavior by upregulating pCREB and PER2 levels in CA1 [50, 52]. The effect of lithium on PER2 levels may involve the inhibition of GSK3β, which promotes PER2 translocation into the nucleus [67–69]. Moreover, Martini et al. demonstrated that knockout of *Per2* in glial cells of nucleus accumbens (NAc) resulted in antidepression-like behavior in mice [21], which is similar to our findings of *Per2* knockdown in CA1 region in this study. In this study, overexpression of *Per2* in CA1 led to depressive-like behavior, and rescued the manic-like behavior induced by ouabain. Moreover, the effect of LV-*Per2* on behavior was more pronounced 12 days after injection than 5 days post injection (Fig. 3E–G, Fig. 3M–O), which may be due to the enhanced expression effect of the lentivirus over time.

Intriguingly, we found that PER2 also positively regulates CREB in a feedback manner (Figs. 2K and 3K). The Co-IP result demonstrated that PER2 co-immunoprecipitated with CREB (Fig. 5M), consistent with a recent study by Brenna et al., who showed that PER2 physically interacts with CREB in the SCN and regulates its binding to the CRE element on *Per1* to induce its expression [70]. Although, we examined different brain region, we propose that PER2 may positively regulate CREB through protein-protein interactions, which requires further investigation.

Currently, most animal models of BD exhibit only one phenotype, primarily focusing on mania-like behavior. It has been demonstrated that ouabain induces a mania-like phenotype one week after ICV injection, which transitions to a depression-like phenotype one week later [47]. In this study, we used the ouabain-induced mania model and lithium, a commonly used treatment, to demonstrate that *Per2* in the rat CA1 region is involved in both the pathogenesis of mania and its treatment. In 2021, Albrecht and colleagues revealed that mice with deletion of *Per2* in glial cells showed reductions in both depression- and impulsivity-like behaviors. In contrast, knockout (KO) of *Per2* in either neurons or glia of the NAc reduced depression-like behavior without affecting impulsivity-like behavior [21]. In comparison, we found that knockdown of *Per2* in CA1 leads to mania-like behavior, characterized by reduced depression- and impulsivity-like behaviors, consistent with their findings of *Per2* KO in glia cells in mice. They also showed that the changes in mood-related behavior were not due to a defective circadian clock, but possibly due to effects on glutamate levels in the NAc [21].

Furthermore, we found that overexpression of *Per2* in CA1 induces depression-like behavior. Therefore, we propose the modulation of *Per2* expression levels in CA1 mediates the transition between mania- and depression-like phenotypes, generating a novel animal model of BD that includes both phenotypes and involves a single gene/factor process, avoiding the two interventions required in other BD models [71].

In conclusion, we found that the CREB–PER2 interaction in CA1 forms a positive feedback loop and mediates the transition between mania- and depression-like behaviors. Knockdown of either *CREB* or *Per2* in CA1 produces mania-like behaviors, while overexpression of either in this region leads to depression-like behaviors. Therefore, modulating the CREB–PER2 loop in CA1 could provide a new animal model of BD. Moreover, this loop may underlie the pathogenesis of the transition between mania and bipolar depression.

## Supplementary information

Supplementary Information

Supplementary Table S1

## Data Availability

All data associated with this study are present in the paper or the supplementary files. Correspondence and requests for the original data and materials should be addressed to Dr. Xin-Ling Wang (wangxinling@email.sdu.edu.cn).
