# Supplementary material for: The crosstalk between CREB and PER2 mediates the transition between mania- and depression-like behavior
Source: Neuropsychopharmacology. 2025 Feb 27;50(11):1683–94. doi: 10.1038/s41386-025-02076-5 (PMC12436625; doi:10.1038/s41386-025-02076-5)
Supplement: Supplementary file 1 — Supplementary Information [file 41386_2025_2076_MOESM1_ESM.pdf]

SUPPLEMENTARY INFORMATION

The crosstalk between CREB and PER2 mediates the transition between mania- and depression-like behavior

Xin-Ling, Wang[1, 2, 3*], Yan-Bin Ji[4], Su-Xia Li[5, 6], Tsvetan Serchov[2, 3*]

Supplementary Figures S1-S7
Supplementary Table S2
Supplementary Results 1-3

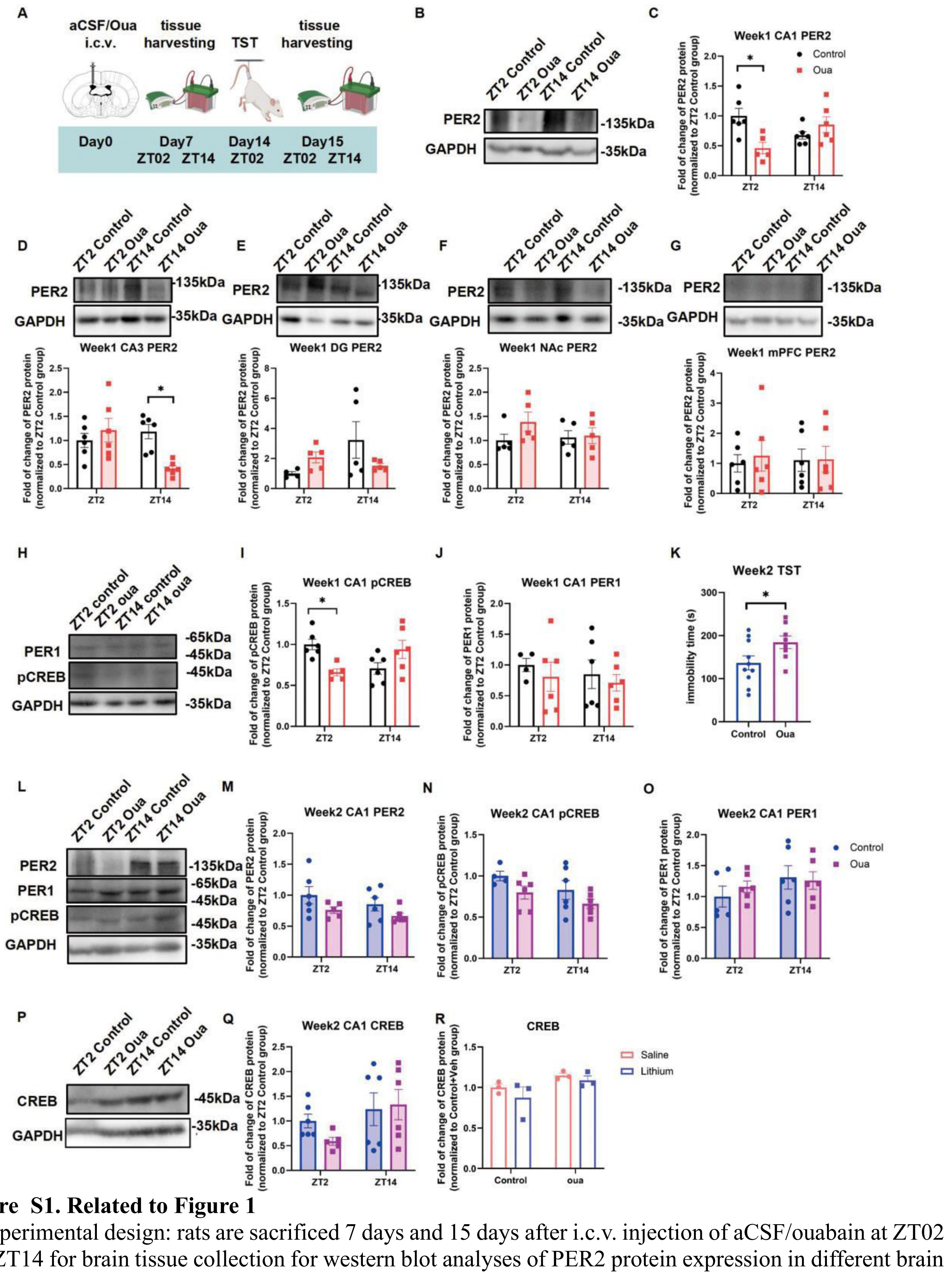

Figure S1. Related to Figure 1

A Experimental design: rats are sacrificed 7 days and 15 days after i.c.v. injection of aCSF/ouabain at ZT02 and ZT14 for brain tissue collection for western blot analyses of PER2 protein expression in different brain regions; on day 14 after Oua injection tail suspension test (TST) was performed. Effects of ouabain on PER2 levels in the CA1 (**B & C**), CA3 (**D**), DG (**E**), NAc (**F**), mPFC (**G**), pCREB (**H & I**) and PER1 (**H & J**) in the CA1 region one week after ouabain model induction (n=5). **K** Immobility time of TST conducted at the second week after ouabain injection (n=10, two-tailed Student's t test). Representative blots (**L & P**) and quantifications of PER2 (**M**), pCREB (**N**), PER1 (**O**) and CREB (**Q**) in CA1 two weeks after ouabain model induction. **R** Quantification of CREB expression in CA1 one week after ouabain model induction and Li treatment (n=5). (two-way ANOVA with Tukey's post-hoc test: $*P < 0.05$). Data are presented as mean ±SEM and the individual data points are depicted. See also Fig. 1 and Table S1. Some of the sketches were made with biorender.com

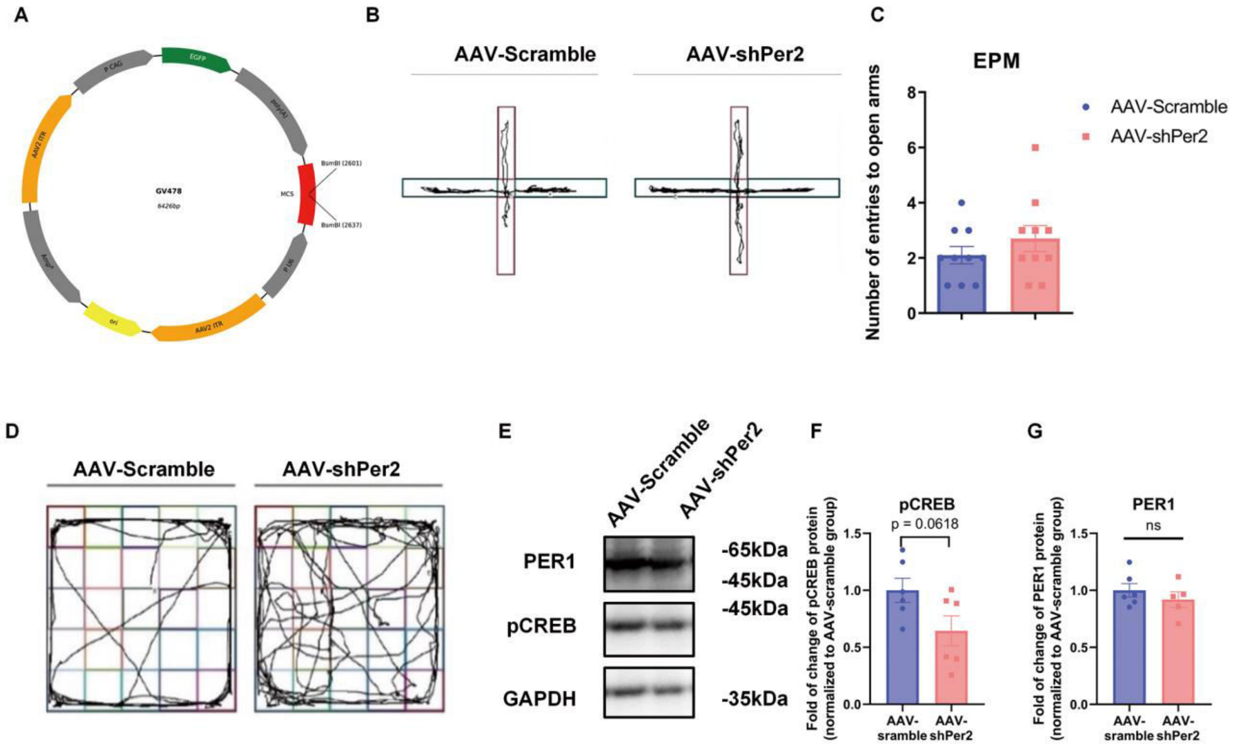

Figure S2. Related to Figure 2

A AAV-pCAG-EGFP-pU6-shPer2/Scramble viral vector map. **B** Representative traces of AAV-shPer2/Scramble injected rats in the EPM. **C** Number of entries into the open arms of EPM (n=10 rats per group, two-tailed Student's t test). **D** Representative traces of AAV-shPer2/Scramble injected rats in the OFT. Representative western blots (**E**) and quantification of pCREB (**F**) and PER1 (**G**) protein levels in CA1 region of AAV-shPer2/Scramble injected rats at ZT02 (n=6 rats per group, two-tailed Student's t test). Data are presented as mean ±SEM and the individual data points are depicted. See also Fig. 2 and Table S1. Some of the sketches were made with biorender.com

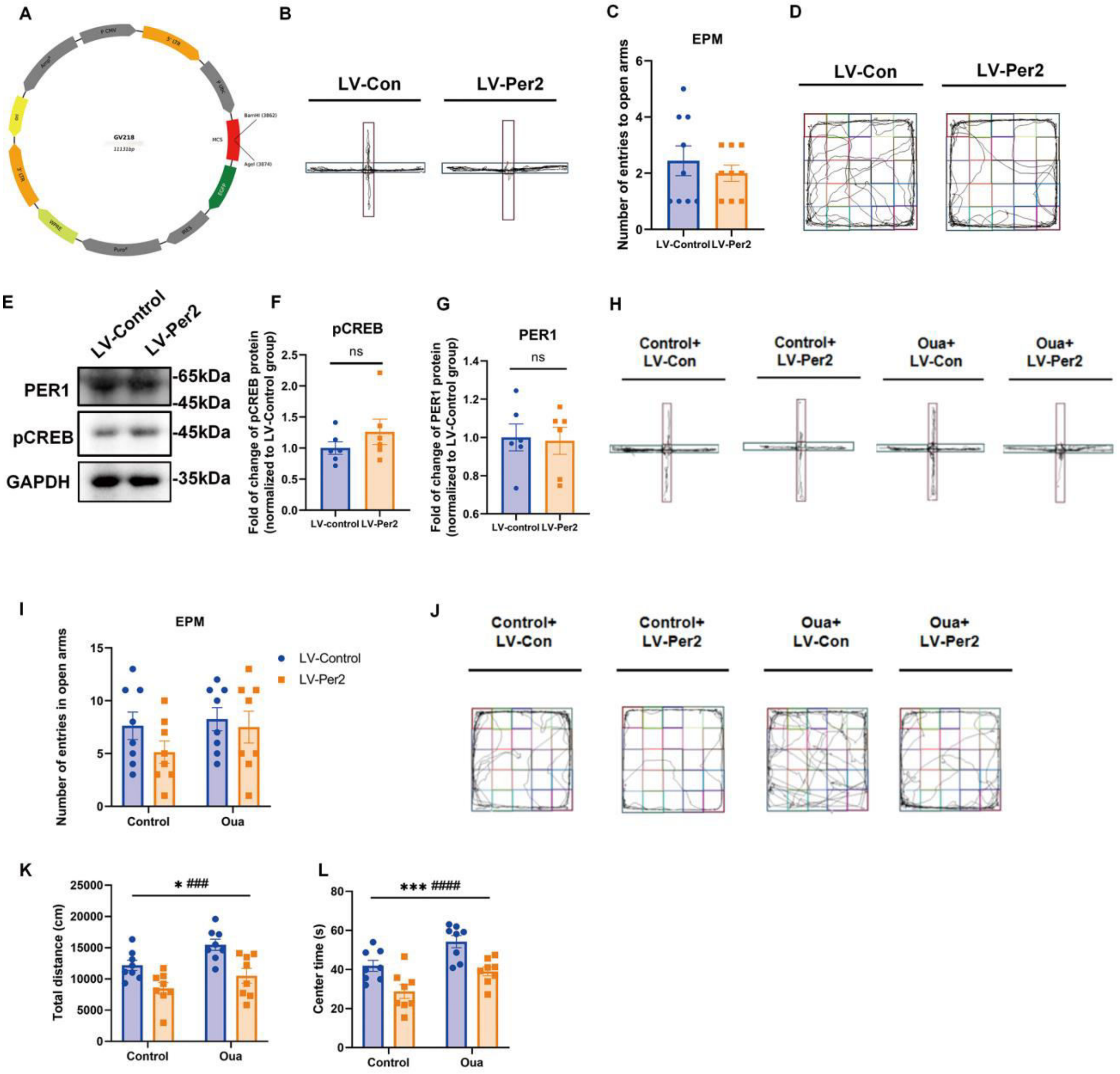

Figure S3. Related to Figure 3.

A LV-CMV-Per2-EGFP viral vector map. **B** Representative traces of LV-Control/Per2 injected rats in EPM test. **C** Number of entries into the open arms in the EPM. **D** Representative traces of LV-Control/Per2 injected rats in the OFT (n=9 rats per group). Representative western blots (**E**) and quantifications of pCREB (**F**) and PER1 (**G**) levels in the CA1 region of LV-Control/Per2 injected rats (n=6 rats per group, two-tailed Student's *t* test). **H** Representative traces of ouabain model rats injected with LV-Control/Per2 in EPM. **I** Number of entries into the open arms in the EPM. **J** Representative traces of ouabain model rats injected with LV-Control/Per2 in OFT. Total distance (**K**) and time spent in the central zone (**L**) of OFT (n=8 rats per group, two-way ANOVA with Tukey's post-hoc test: *$P < 0.05$, ***$P < 0.001$, compared with the control + LV-control group; ###$P < 0.001$, ####$P < 0.0001$, compared with the ouabain + LV-control group. Data are presented as mean ±SEM and the individual data points are depicted. See also Fig. 3 and Table S1. Some of the sketches were made with biorender.com

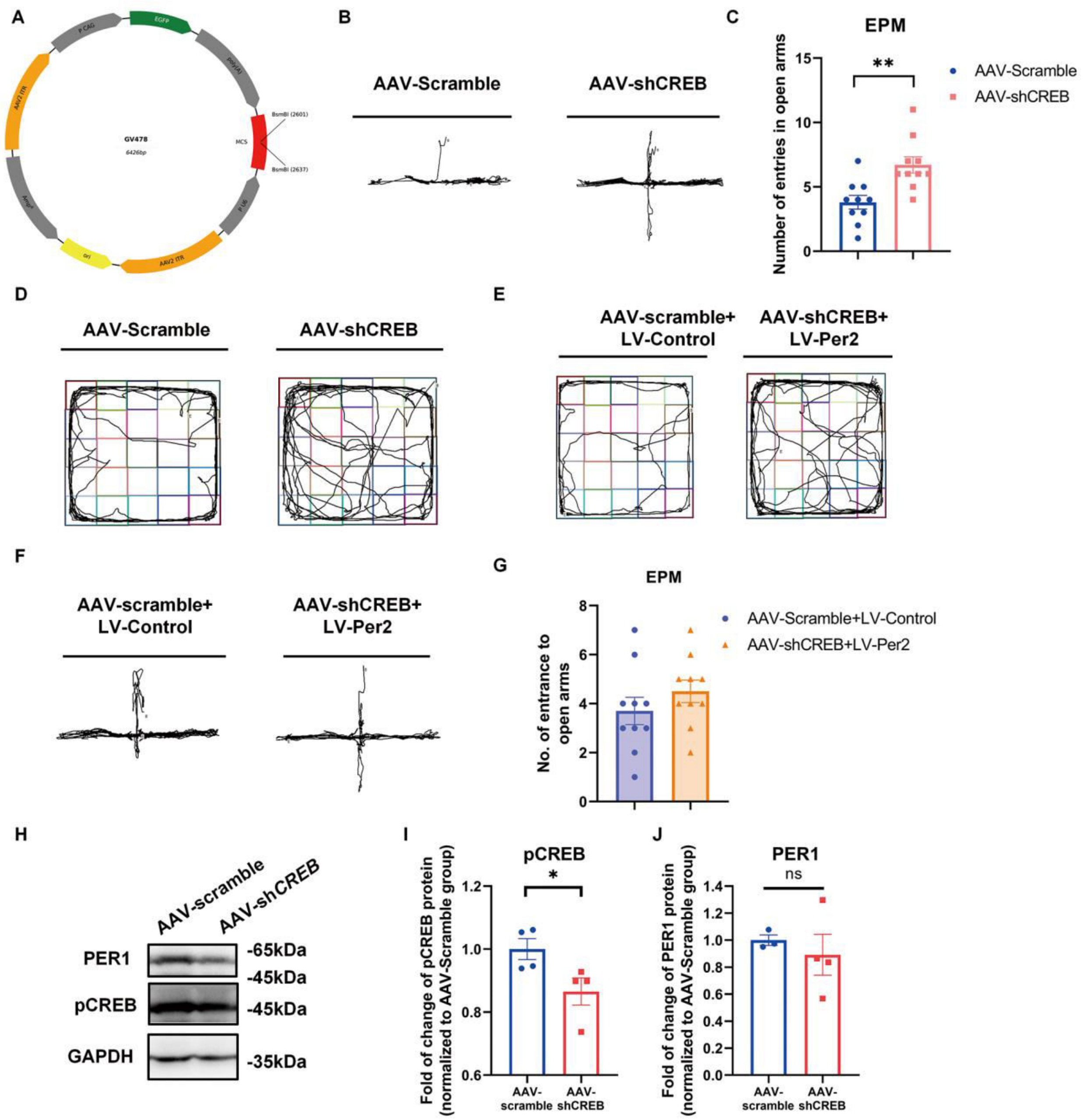

Figure S4. Related to Figure 4.

A AAV-pCAG-EGFP-pU6-shCREB/Scramble viral vector map. **B** Representative traces of AAV-Scramble/shCREB injected rats in EPM. **C** Number of entries into the open arms of EPM (n=10). **D** Representative traces of AAV-Scramble/shCREB injected rats in OFT. Representative traces of AAV-Scramble/shCREB and LV-Control/Per2 injected rats in OFT (**E**) and EPM (**F**). **G** Number of entries into the open arms of EPM (n=10). Representative western blots (**H**) and quantification of pCREB (**I**) and PER1 (**J**) levels in the CA1 region of AAV-Scramble/shCREB injected rats (n=4, two-tailed Student's t test: *$P <$ 0.05, **$P < 0.01$). Data are presented as mean ±SEM and the individual data points are depicted. See also Fig. 4 and Table S1. Some of the sketches were made with biorender.com

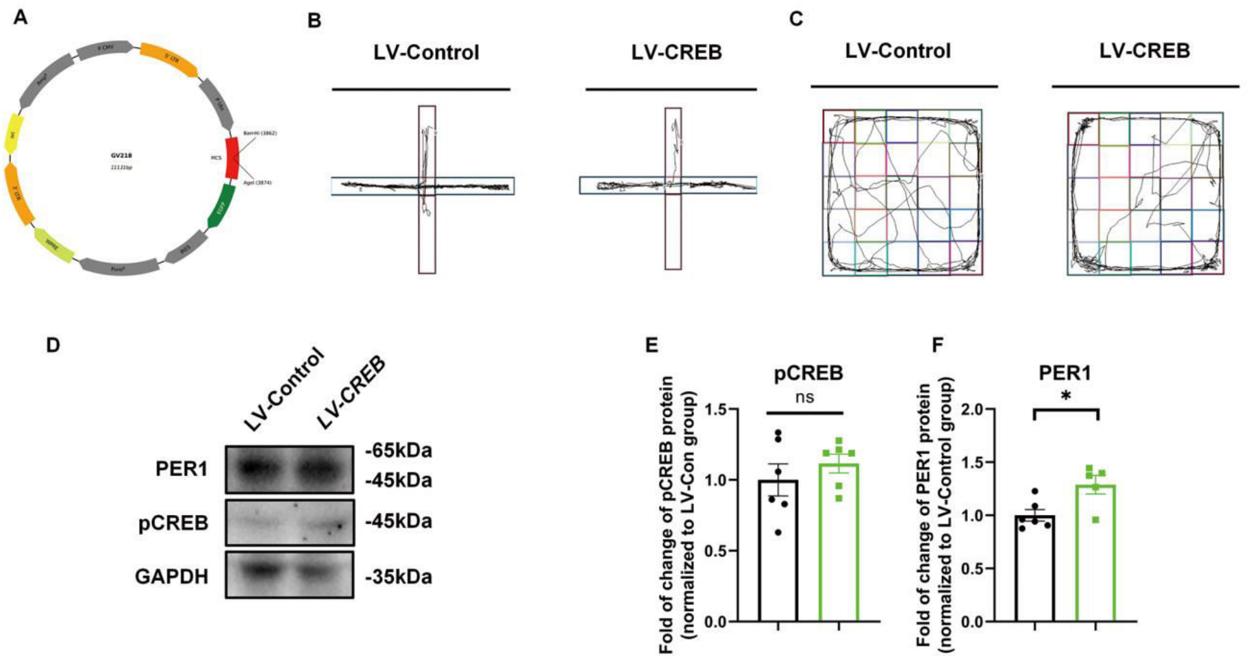

Figure S5. Related to Figure 5.
A LV-CMV-Per2-EGFP viral vector map. **B** Representative traces of LV-Control/CREB injected rats in EPM. **C** Representative traces of LV-Control/CREB injected rats in OFT. Representative western blots (**D**) and quantification of pCREB (**E**) and PER1 (**F**) levels in the CA1 regions of the LV-Control/CREB injected rats (n=6, two-tailed Student's t test: *P < 0.05). Data are presented as mean ±SEM and the individual data points are depicted. See also Fig. 4 and Table S1. Some of the sketches were made with biorender.com

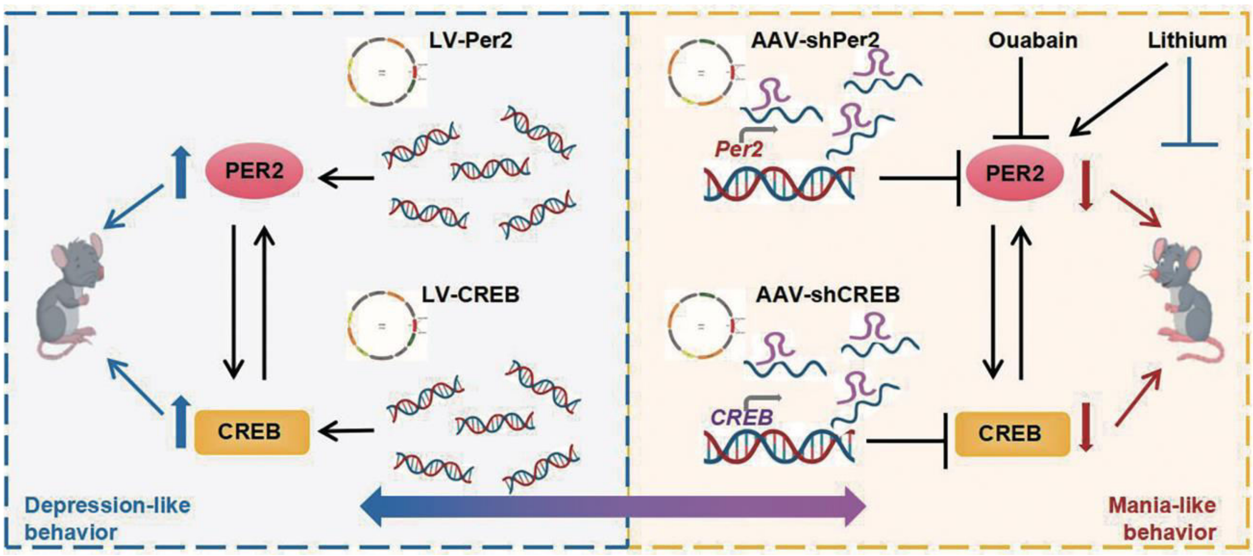

Figure S6. Crosstalk between CREB and PER2 regulates the depression- and mania-like behavior.
Ouabain induces mania-like behavior and downregulates PER2 expression in CA1 hippocampal area. Knockdown of *Per2* in the CA1 region leads to mania-like behaviors and downregulates CREB levels. Similarly, knockdown of CREB in this region also results in mania-like behaviors and decreases PER2 levels. Conversely, lithium exerts anti-mania-like effects by upregulating PER2. Overexpression of *Per2* in the CA1 region induces depression-like behaviors and upregulates CREB levels, while overexpression of CREB produces the same effect as *Per2*, elevating PER2 levels. Thus, CREB and PER2 form a positive feedback loop, and regulations of their levels mediates the transition between mania- and depression-like behaviors.

Figure S7. Full length blots of the representative pictures from the main figures.

Figure1J **GAPDH**

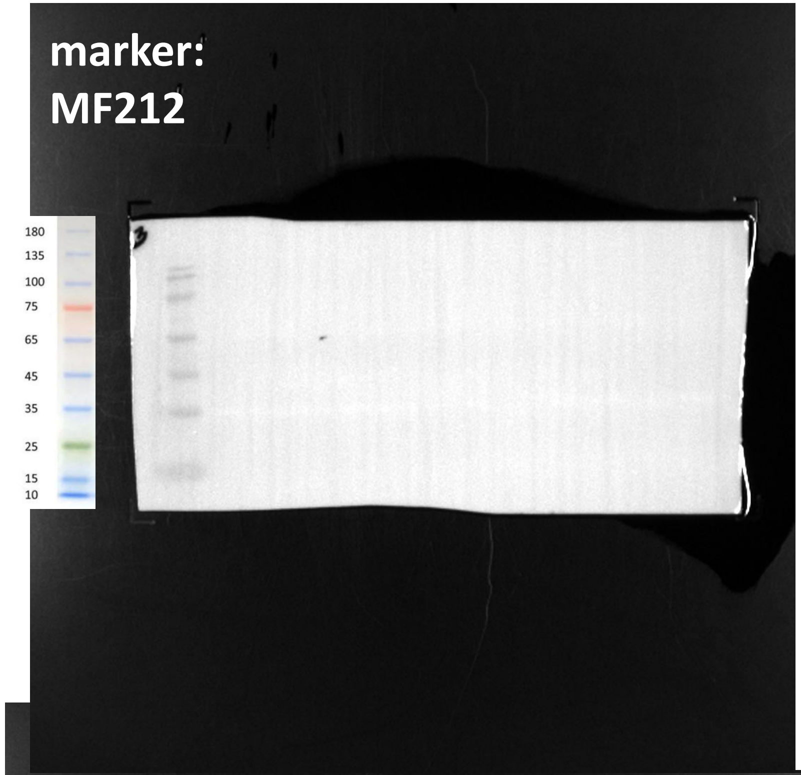

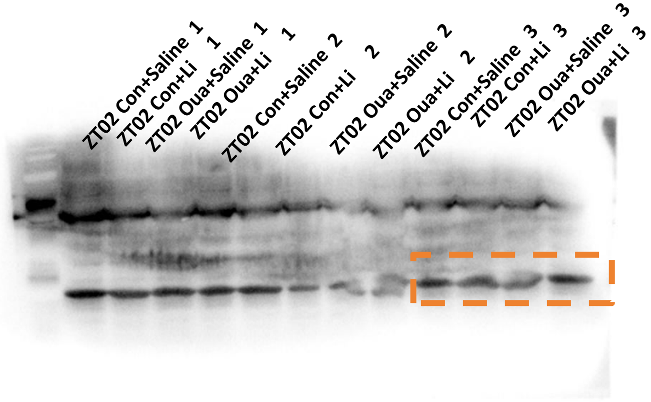

Figure1JK **PER2** No. 3 Membrane

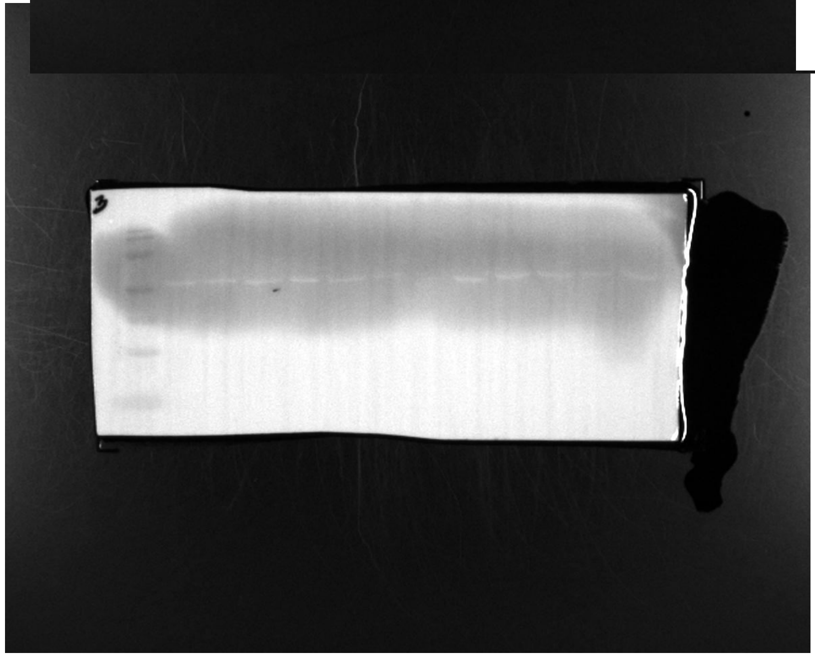

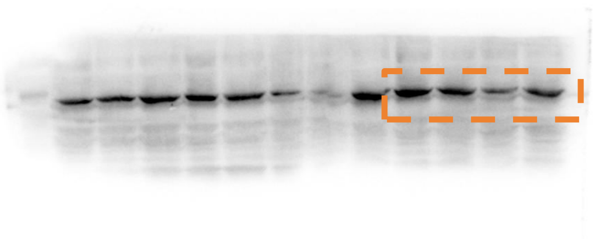

Figure 1J, L **pCREB** No. 3 Membrane

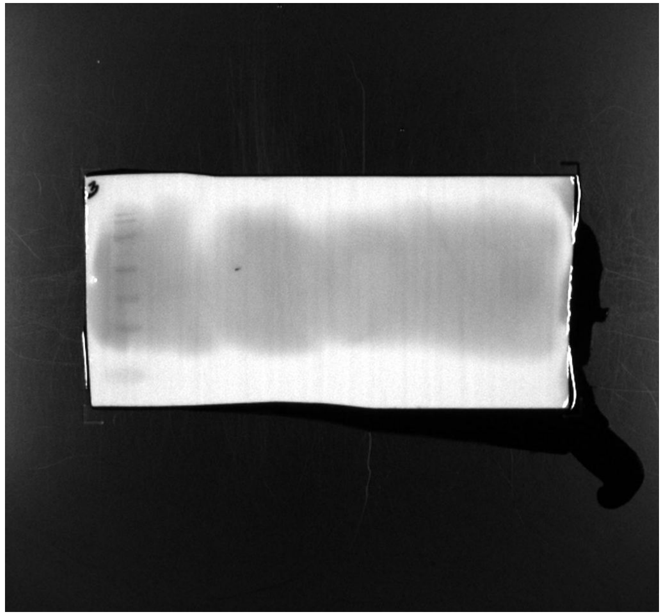

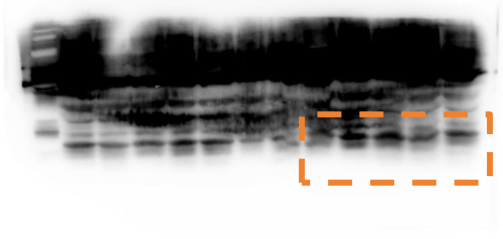

Figure 1J, L **CREB** No. 3 Membrane

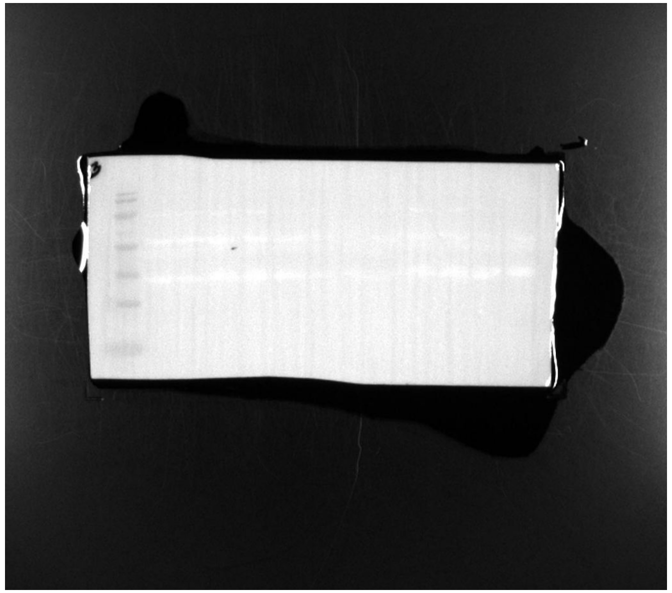

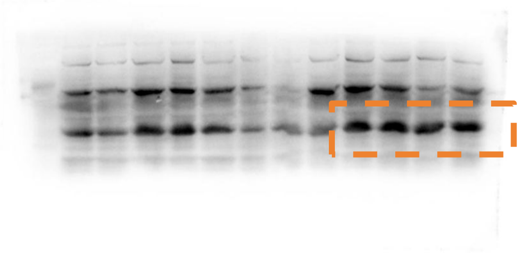

Figure2C GAPDH

marker:
MF212

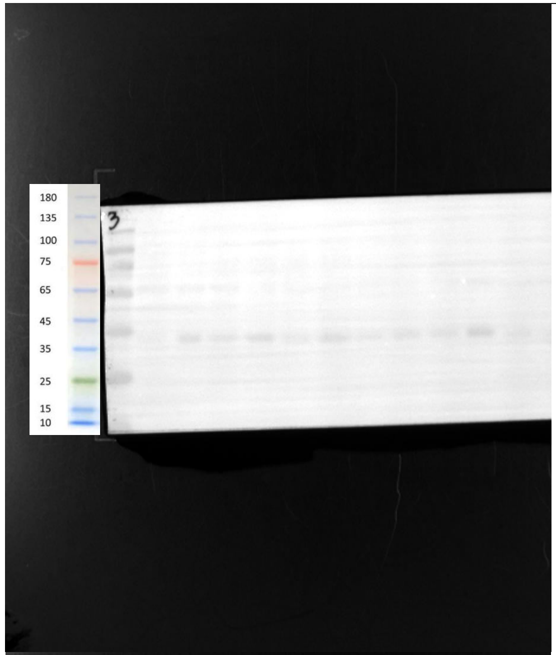

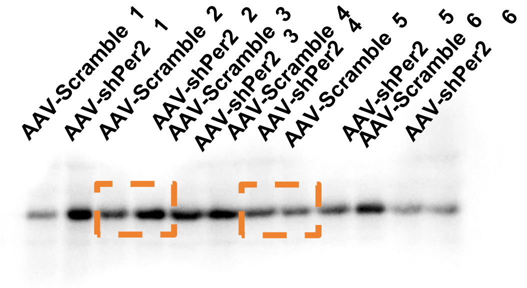

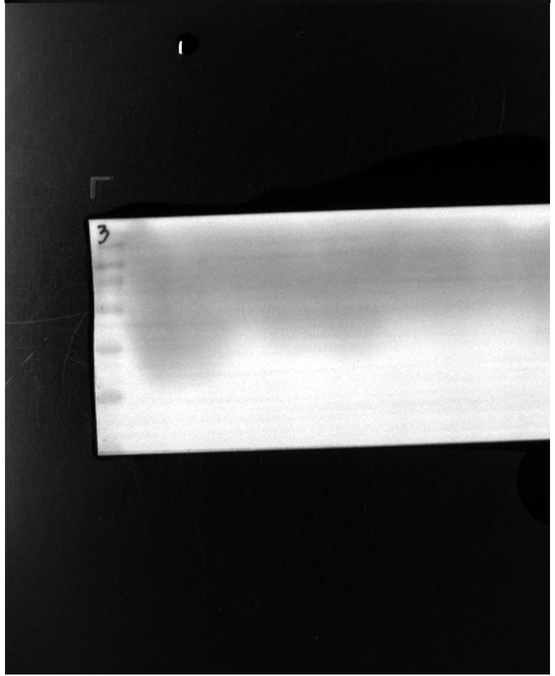

Figure2C, D **PER2**

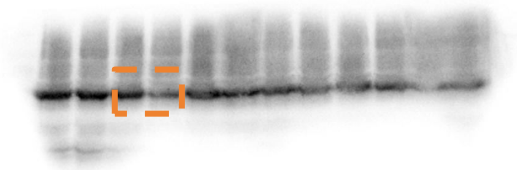

The sequence is the same as GAPDH

FigureS2E, G **PER1**

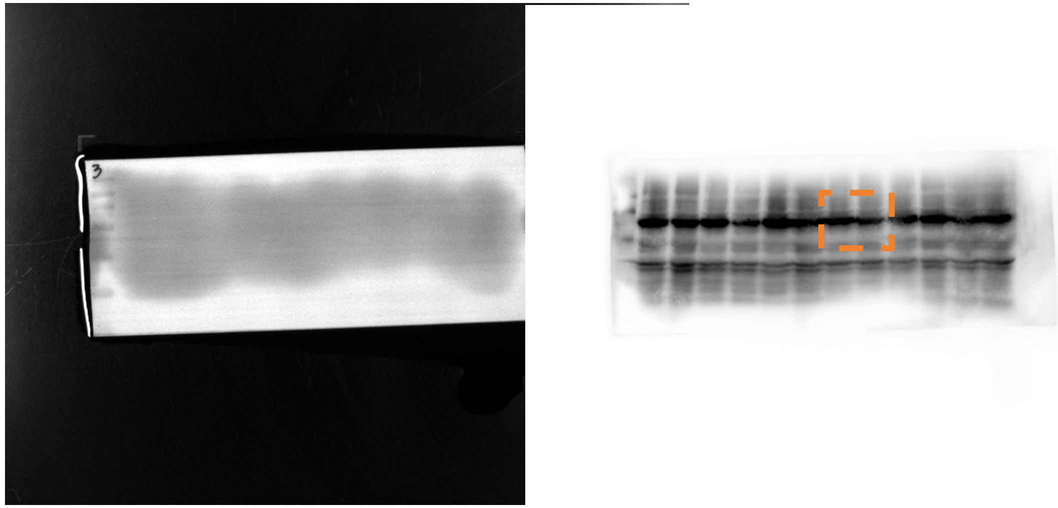

Figure2E, F CREB

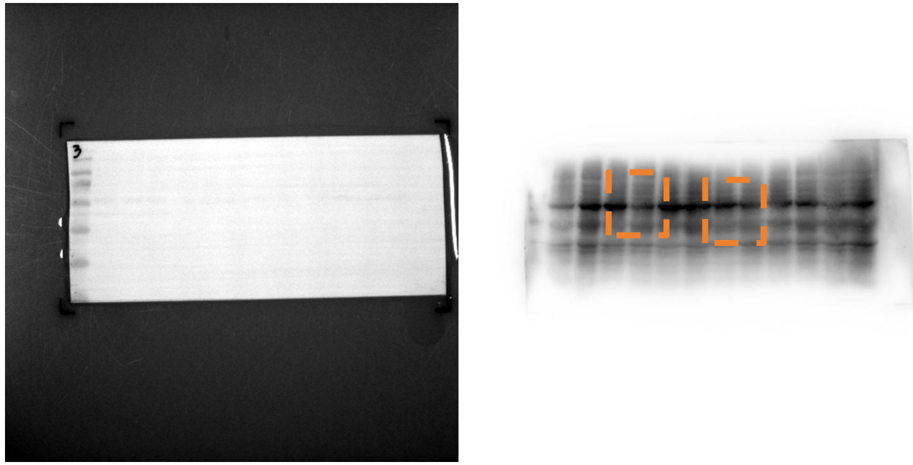

FigureS2E, F pCREB

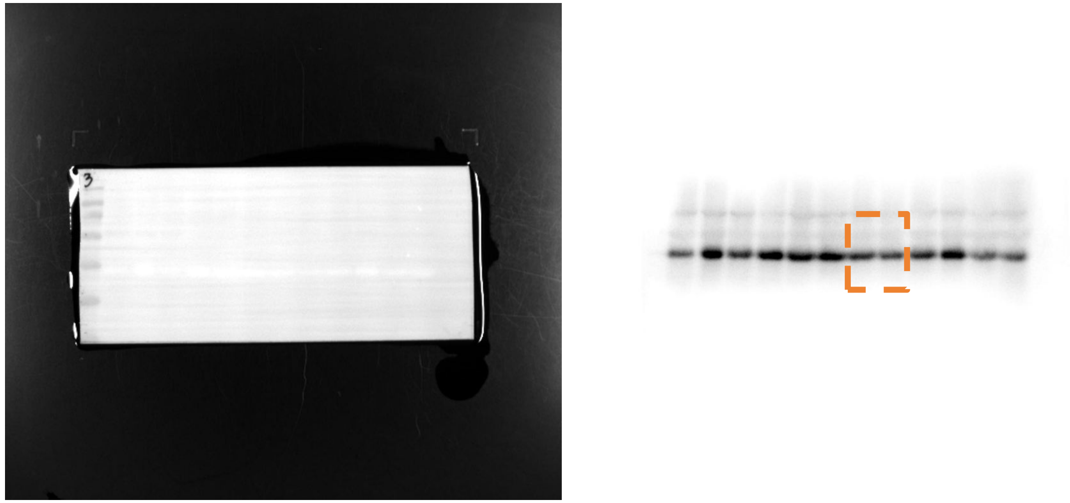

The sequence is the same as GAPDH

marker:
MF212

Figure3C GAPDH

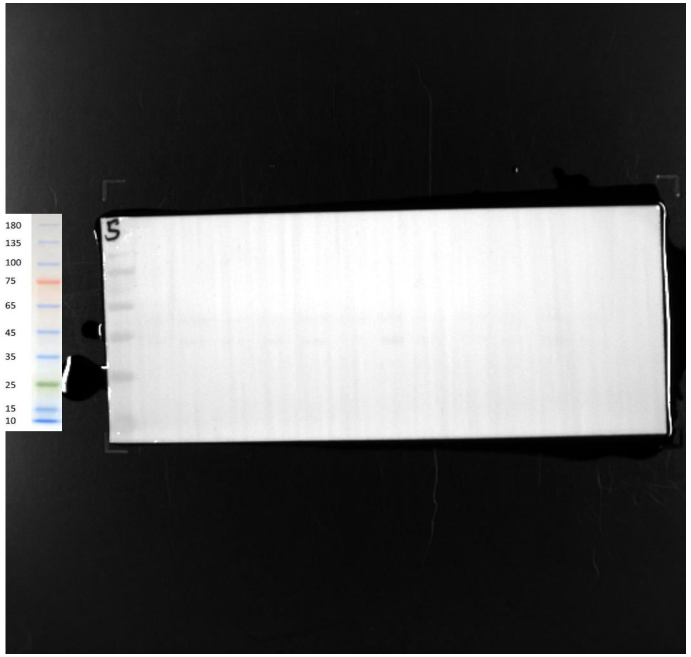

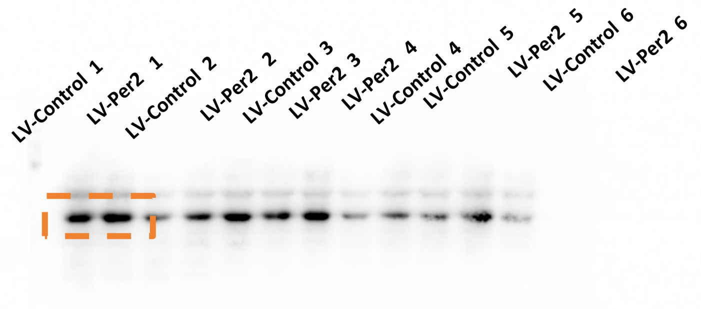

Figure3C,D PER2

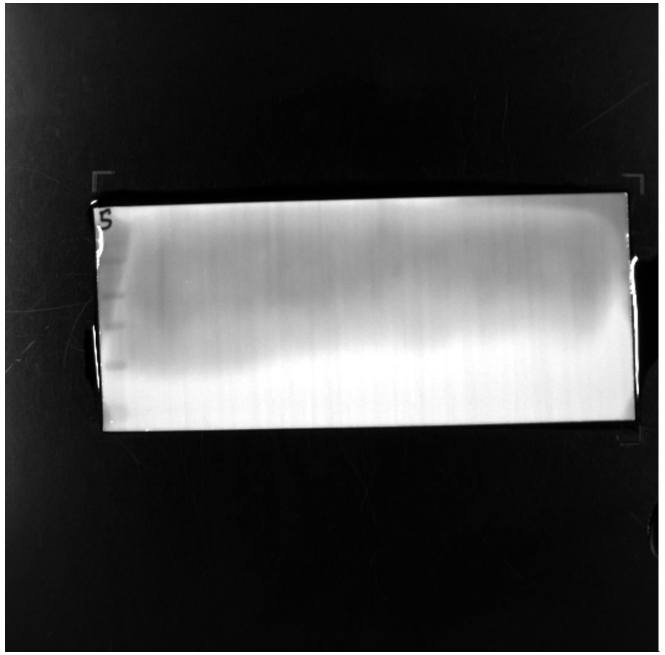

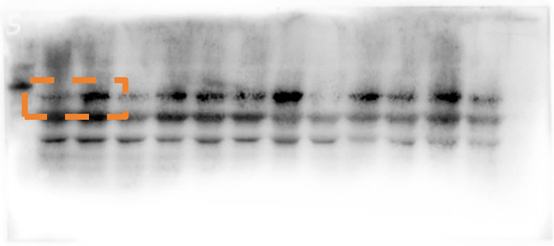

The sequence is the same as GAPDH

Figure3J, K CREB

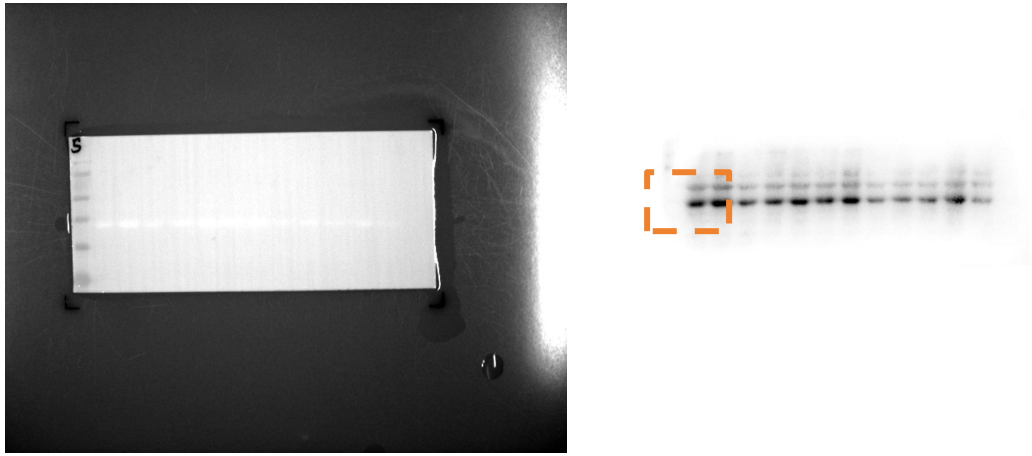

Figure S3E,F pCREB

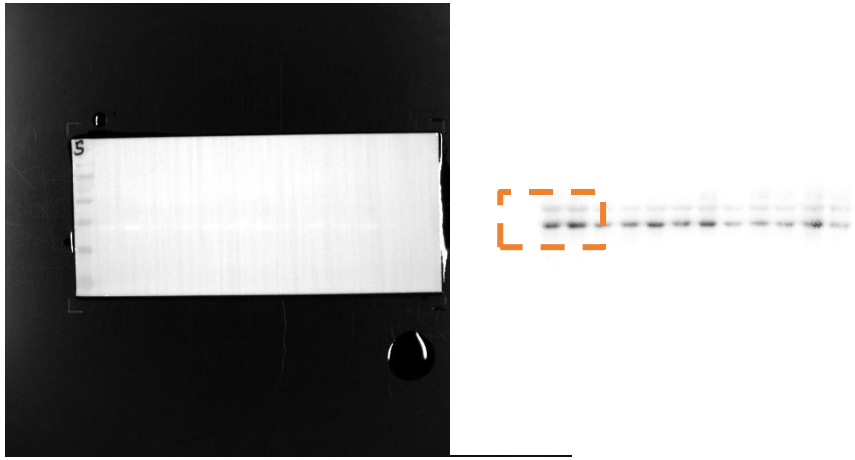

FigureS3EG PER1

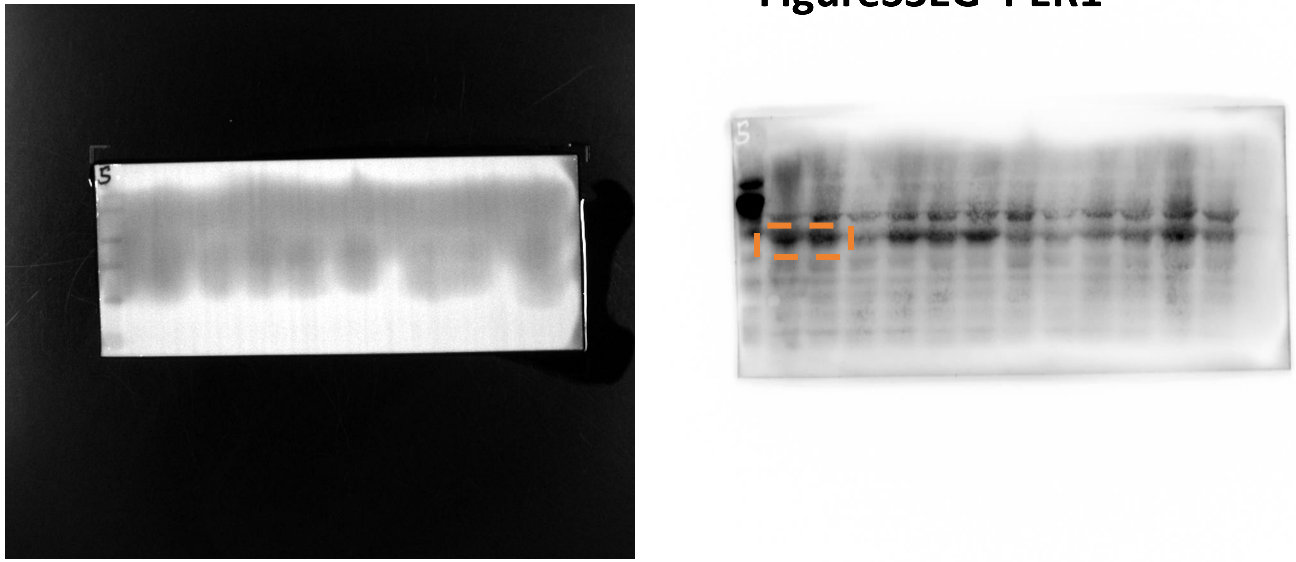

The sequence is the same as GAPDH

Figure3C GAPDH

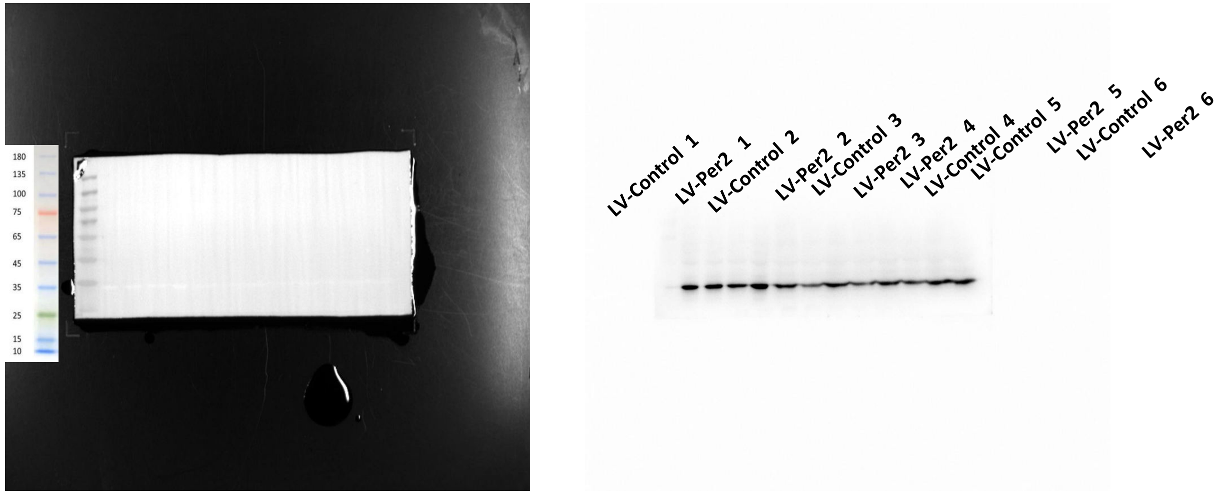

Figure3C, D PER2

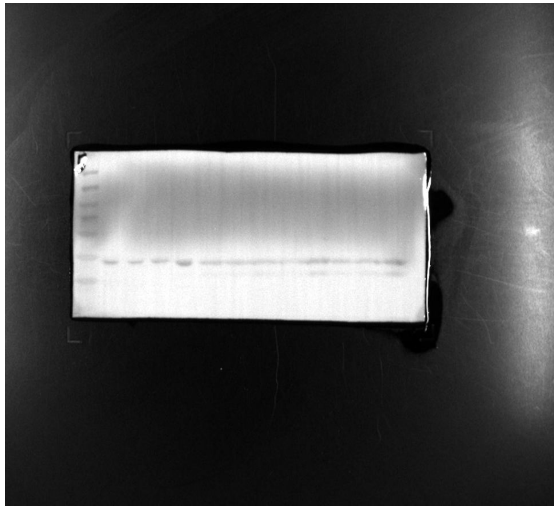

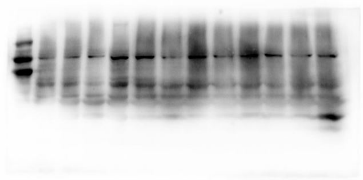

The sequence is the same as GAPDH

Figure3J, K CREB

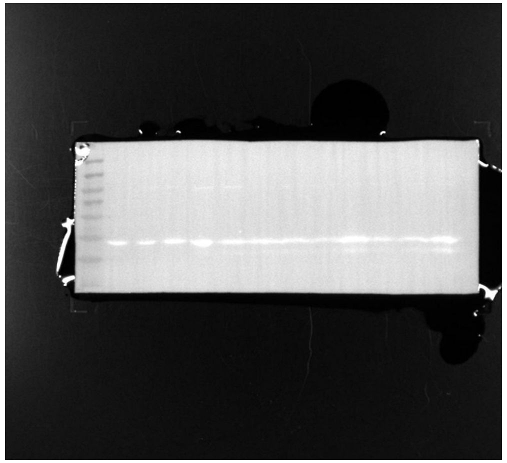

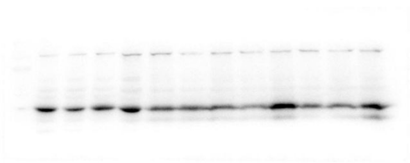

Figure S3E, F pCREB

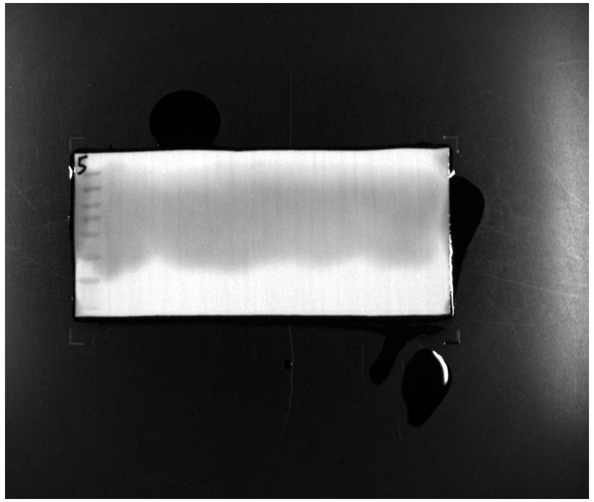

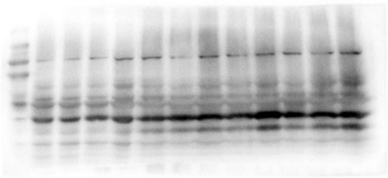

FigureS3E, G PER1

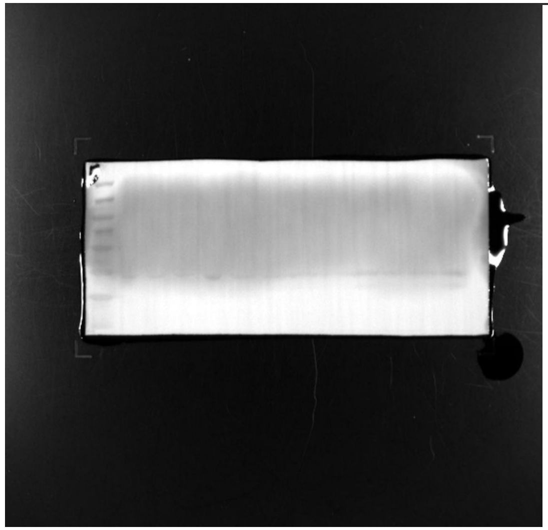

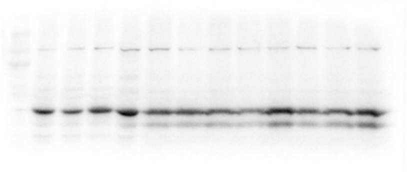

The sequence is the same as GAPDH

Figure4C GAPDH

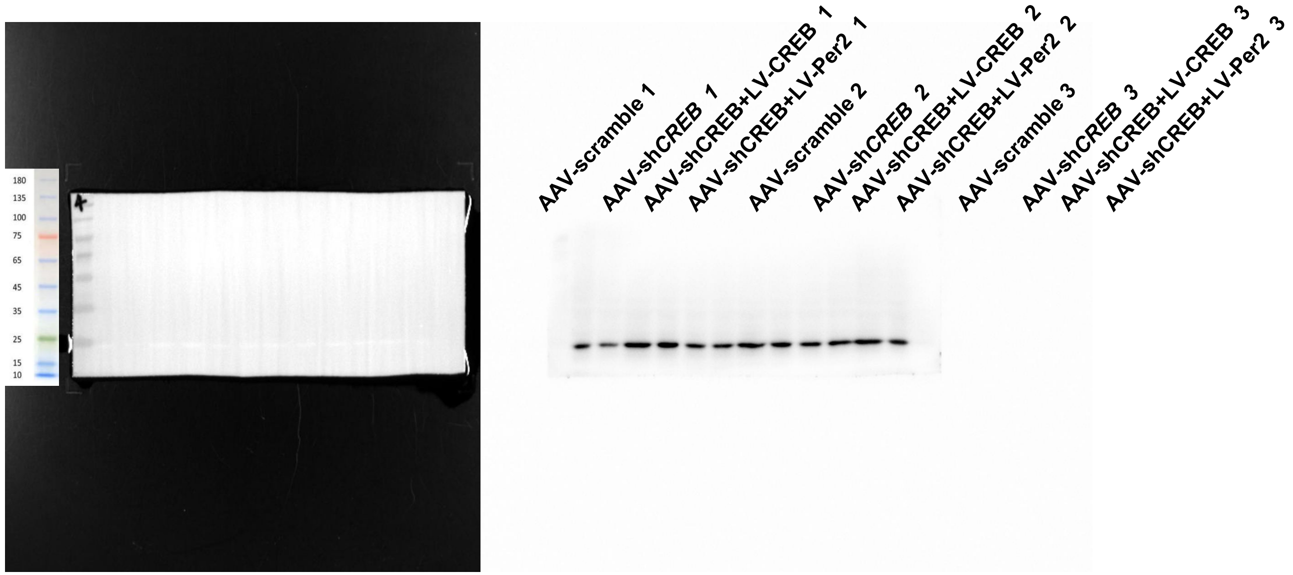

Figure4J, K PER2

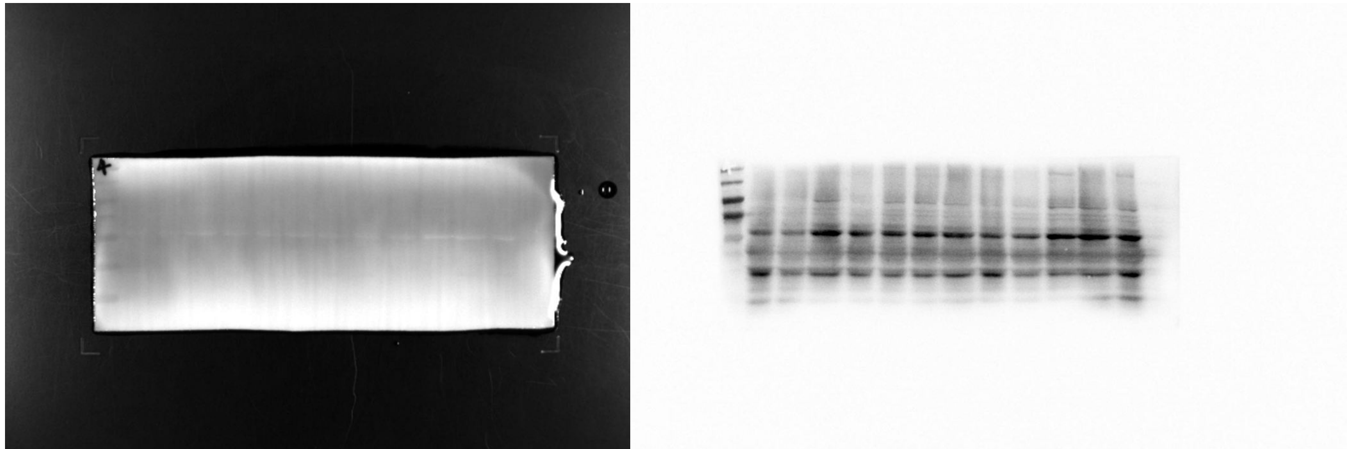

The sequence is the same as GAPDH

Figure4C, D   CREB

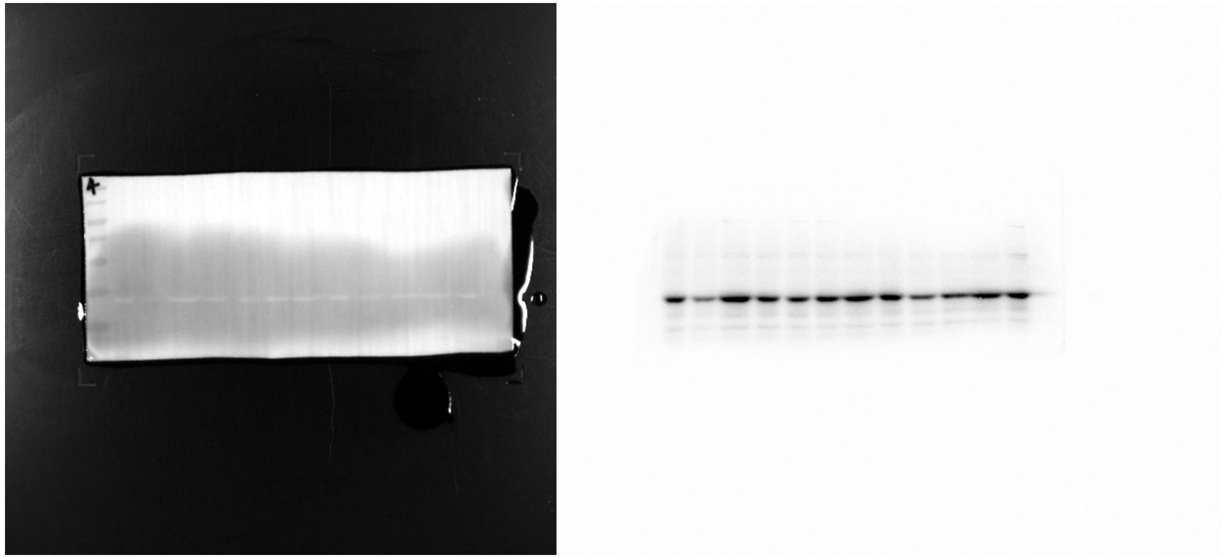

FigureS4H, I   pCREB

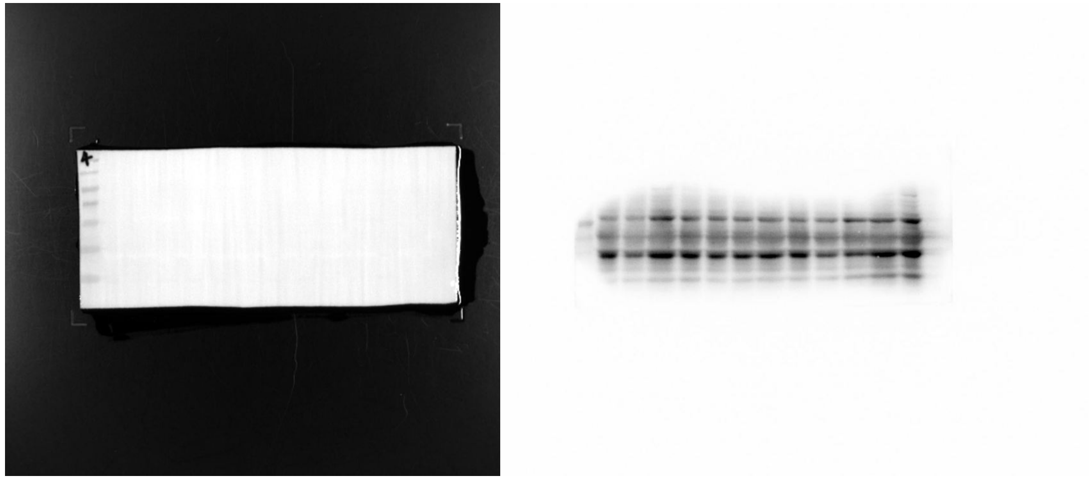

Figure4H, J

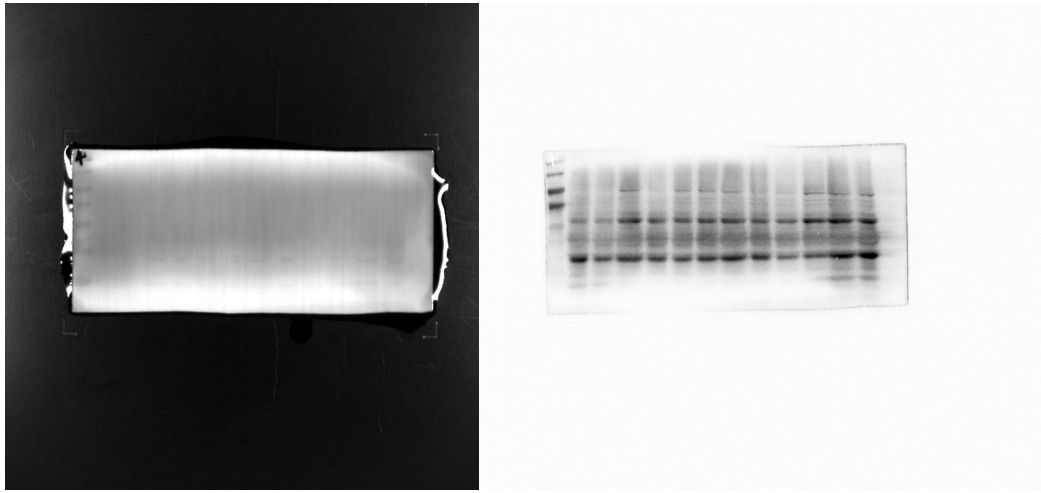

The sequence is the same as GAPDH

Figure4C GAPDH

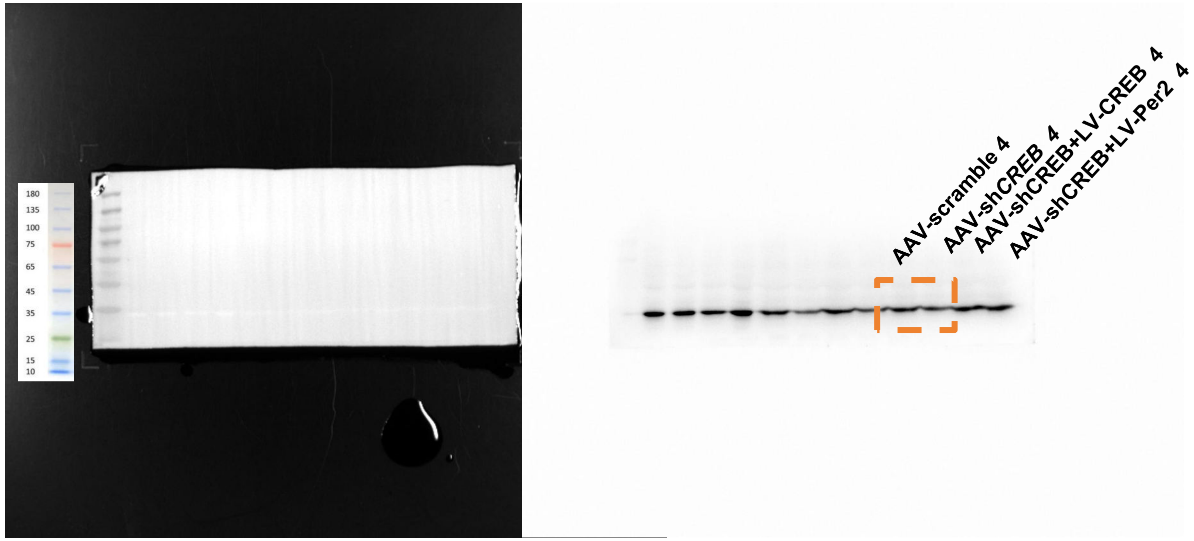

Figure4J, K PER2

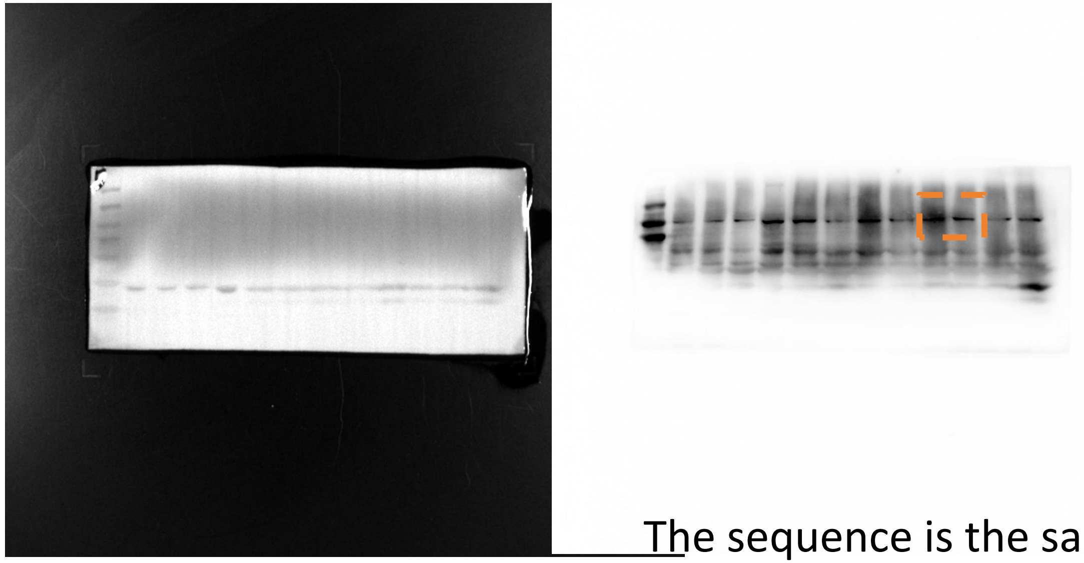

The sequence is the same as GAPDH

Figure4C, D   CREB

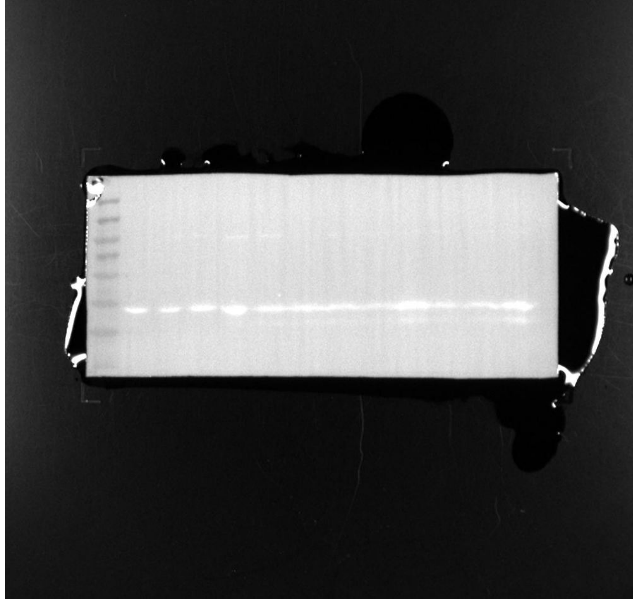

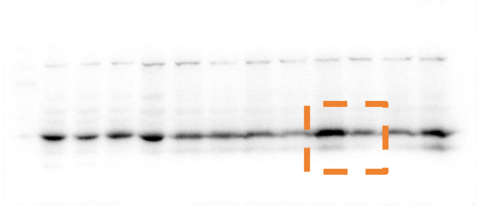

FigureS4E, F   pCREB

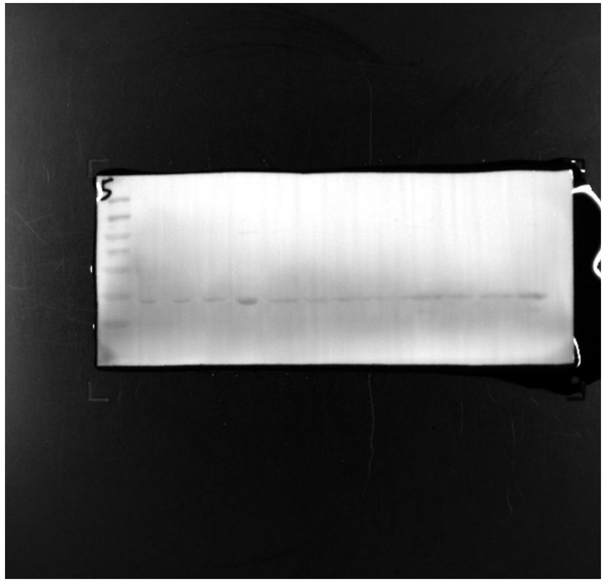

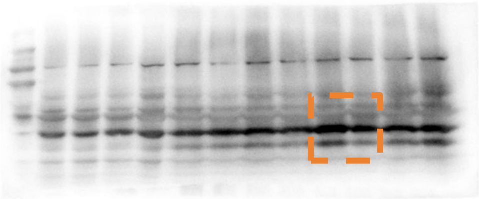

FigureS4E, G PER1

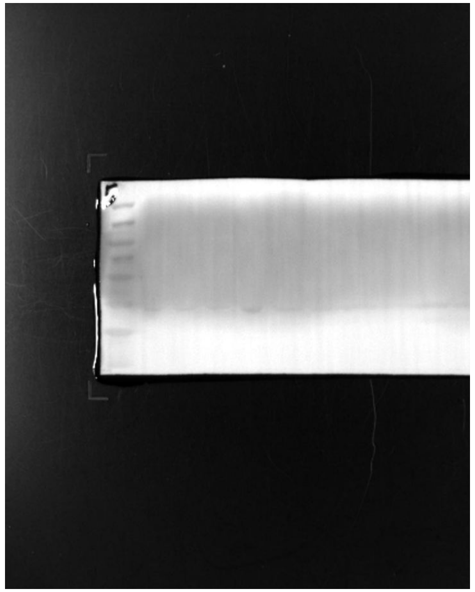

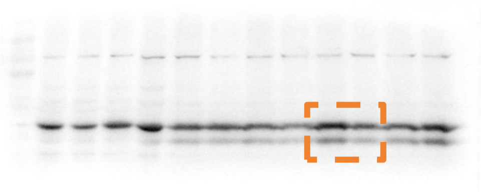

The sequence is the same as GAPDH

Figure5C GAPDH

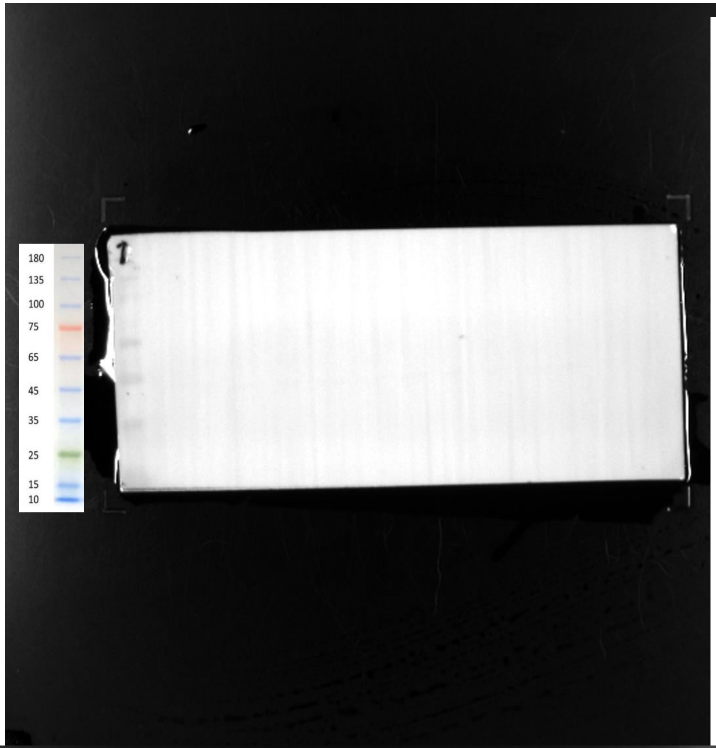

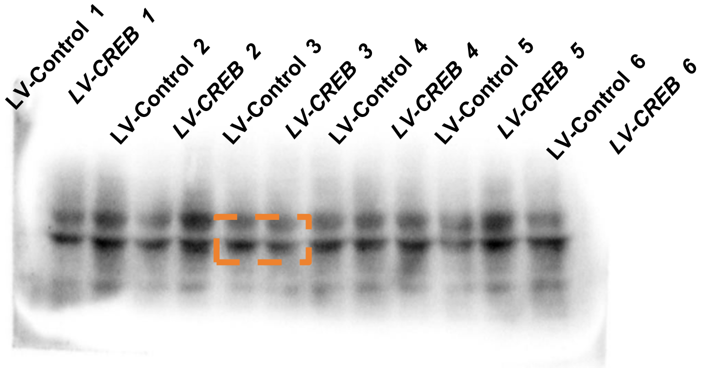

Figure5K, L PER2

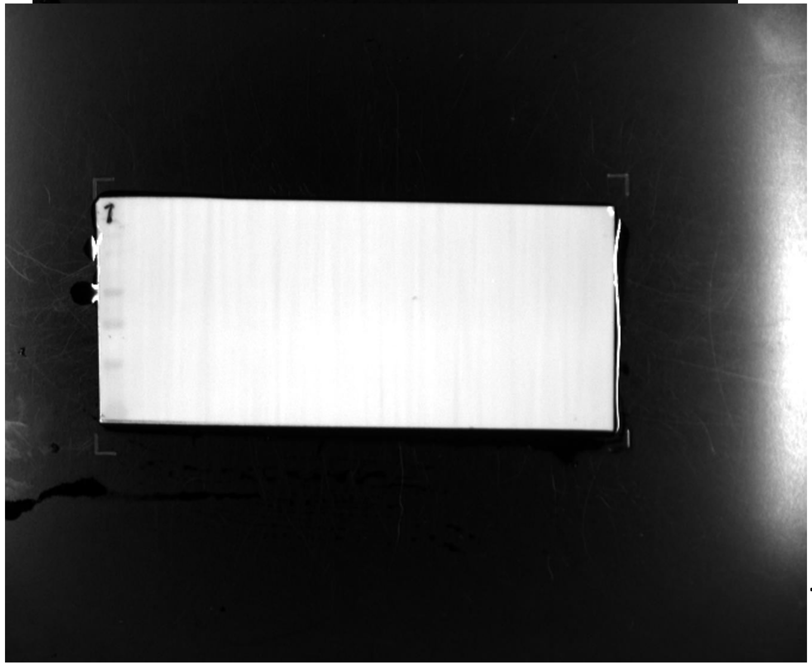

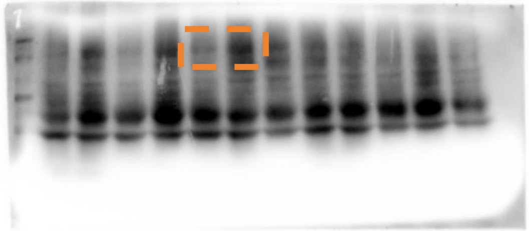

The sequence is the same as GAPDH

Figure5C, D CREB

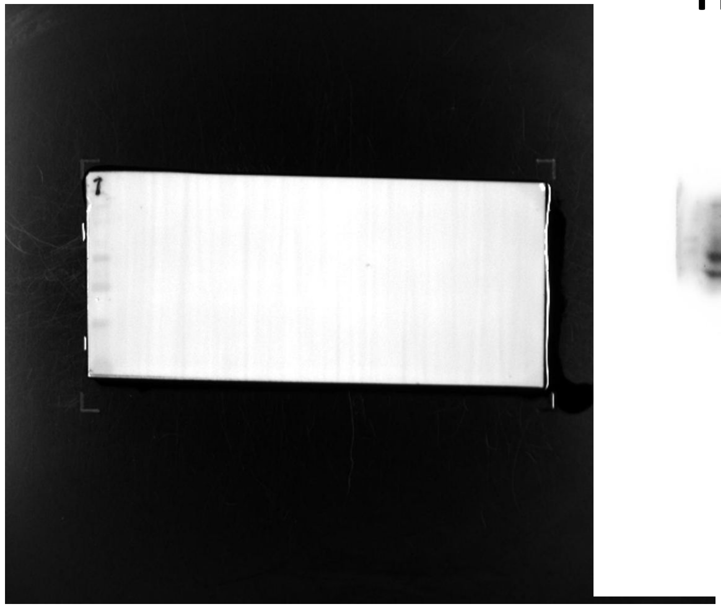

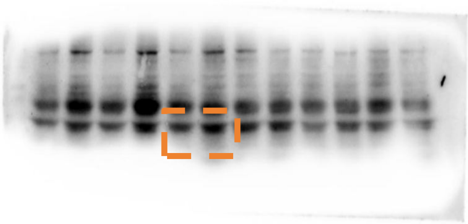

FigureS5D, E

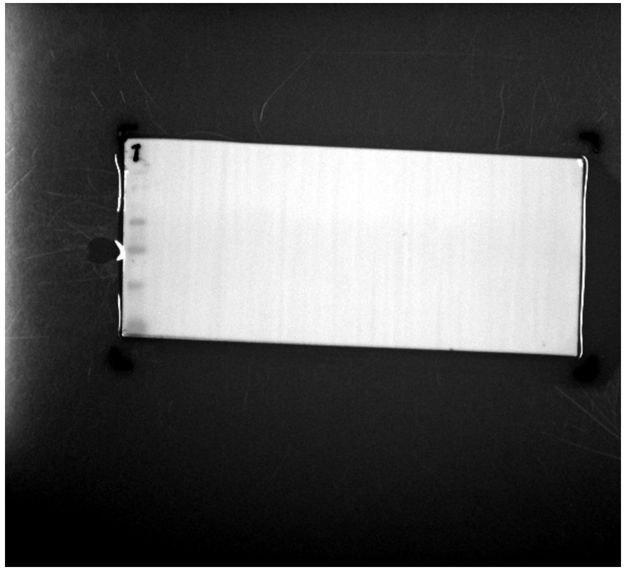

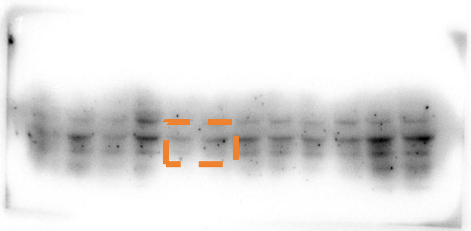

Same as the sequence of GAPDH

FigureS5D, F PER1

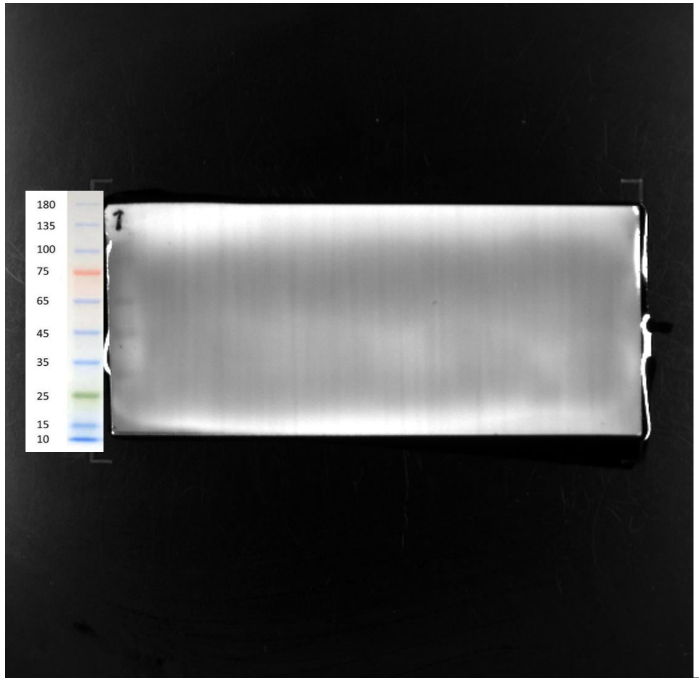

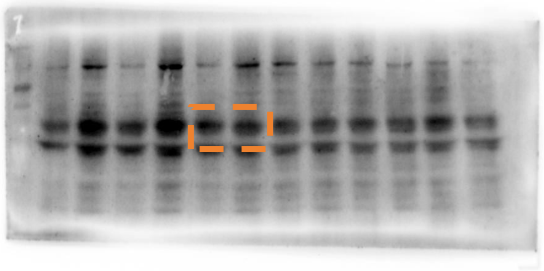

Same as the sequence of GAPDH

FigureS1 CA1 (Week1) GAPDH
No.3 small and No.4 small (molecules)

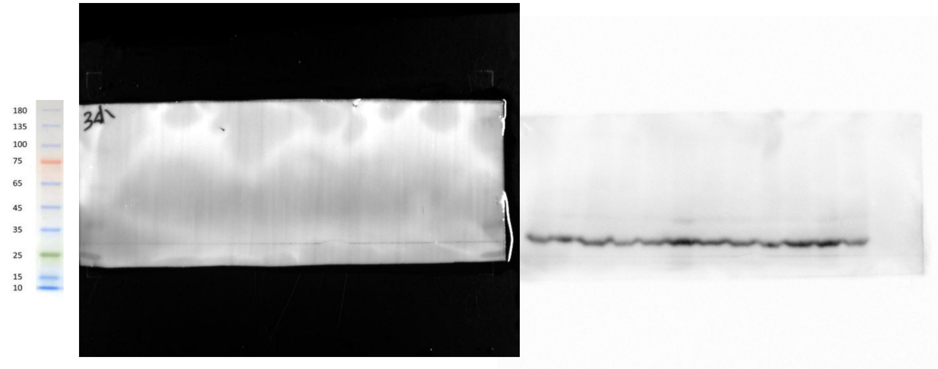

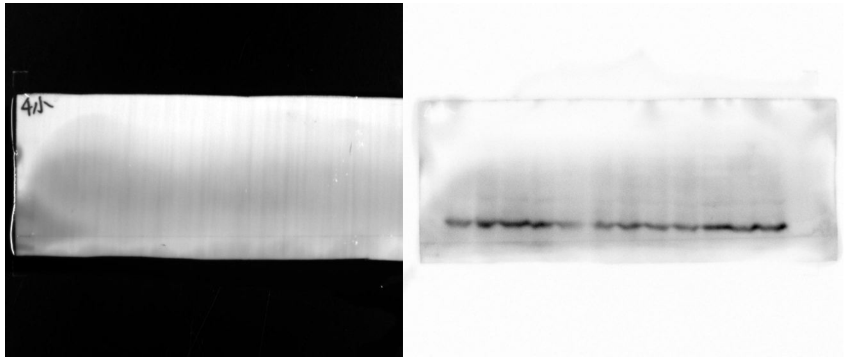

FigureS1 CA1 (Week1) PER2
No.3 large and 4 large (molecules)

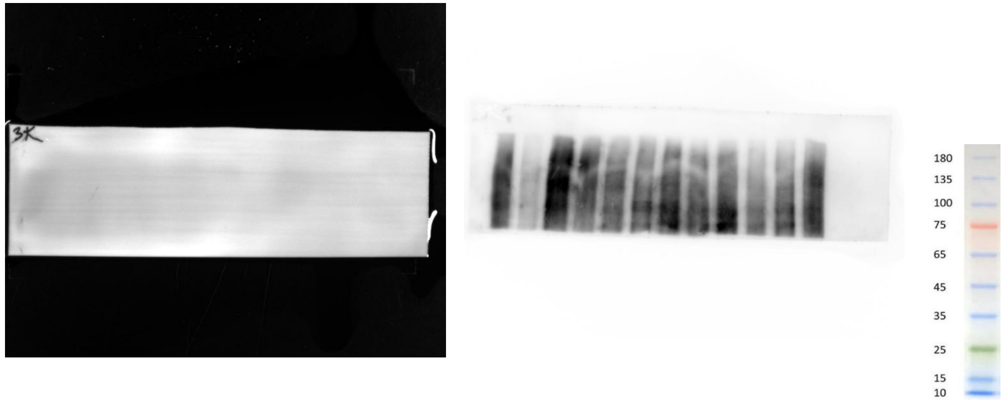

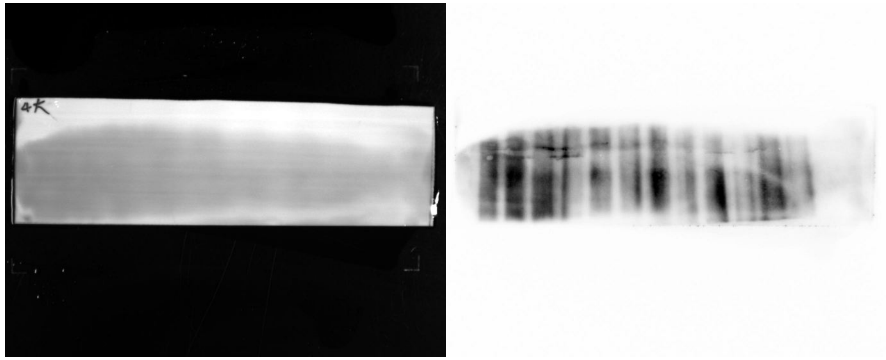

membrane cut at 75kDa, in the sequence of ZT2 control, ZT2 oua, ZT14 Control, ZT14 Oua

FigureS1 CA3 (Week1) GAPDH
No.1 small and No.2 small (molecules)

FigureS1 CA3 (Week1) PER2
No.1 and 2 large (molecules)

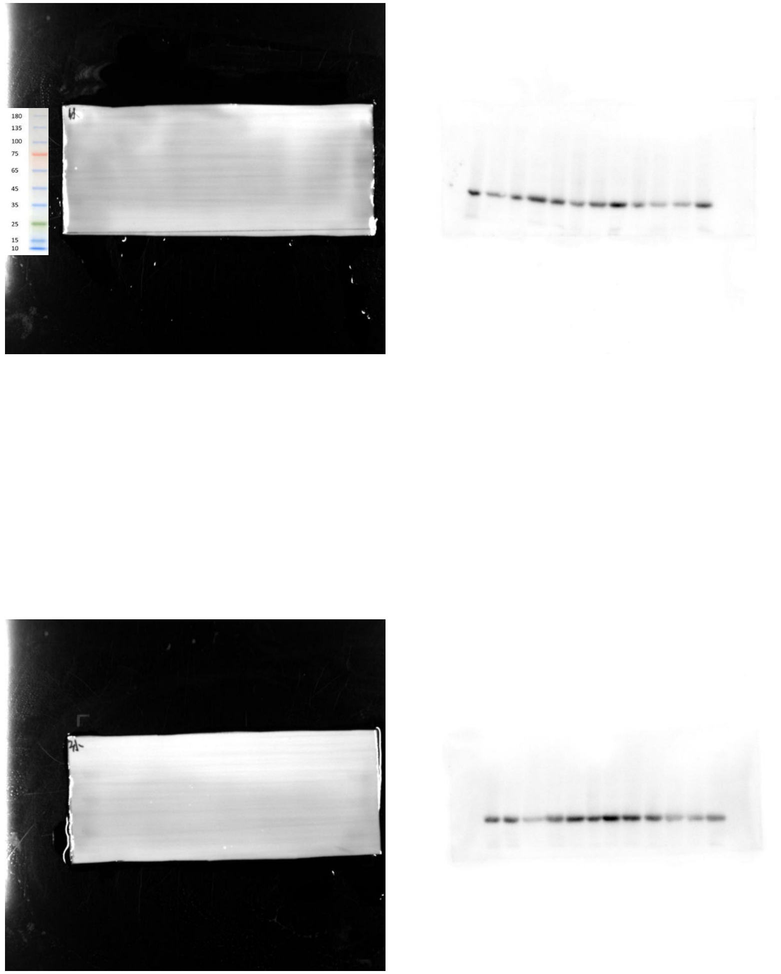

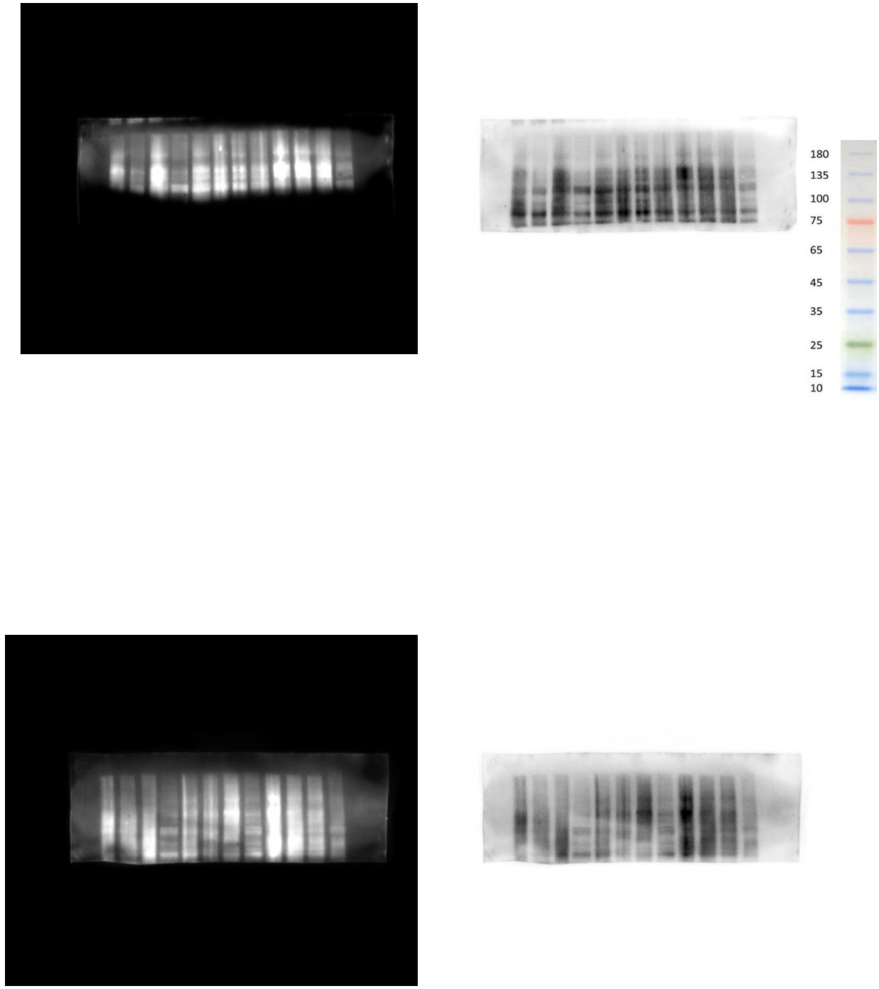

membrane cut at 75kDa, in the sequence of ZT2 control, ZT2 oua, ZT14 Control, ZT14 Oua

FigureS1 DG (Week1) GAPDH
No.3 small and No.4 small (molecules)

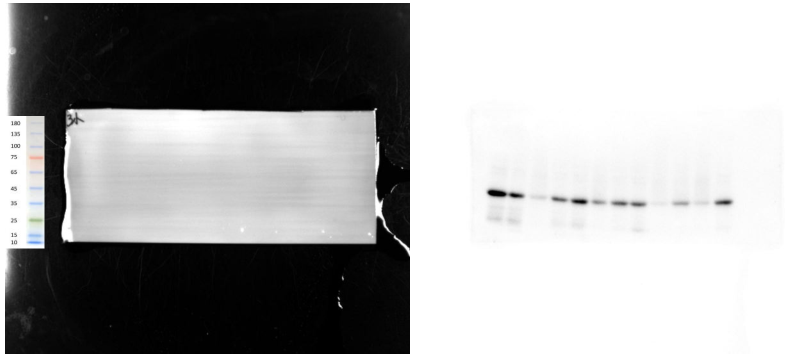

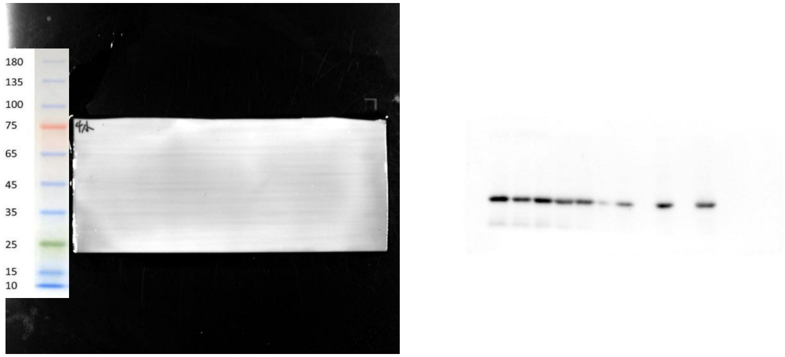

FigureS1 DG (Week1) PER2
No.3 and 4 large (molecules)

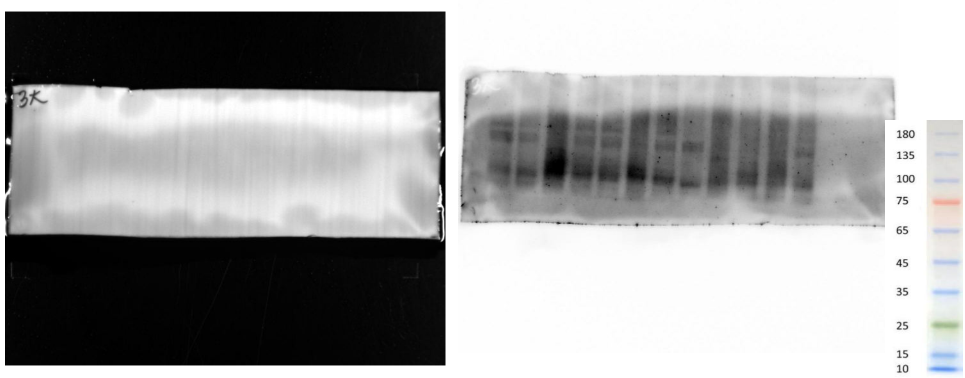

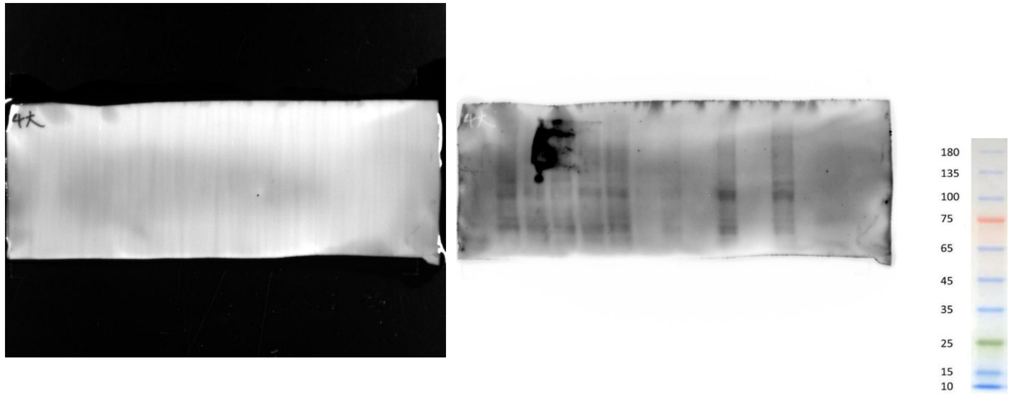

membrane cut at 75kDa, in the sequence of ZT2 control, ZT2 oua, ZT14 Control, ZT14 Oua

FigureS1 mPFC (Week1) GAPDH
No.1 small and No.2 small (molecules)

FigureS1 mPFC (Week1) PER2
No.1 and 2 large (molecules)

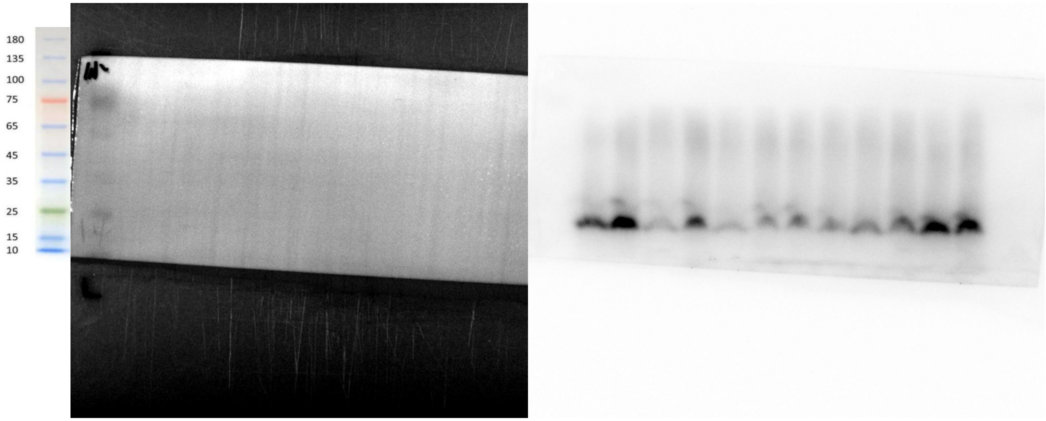

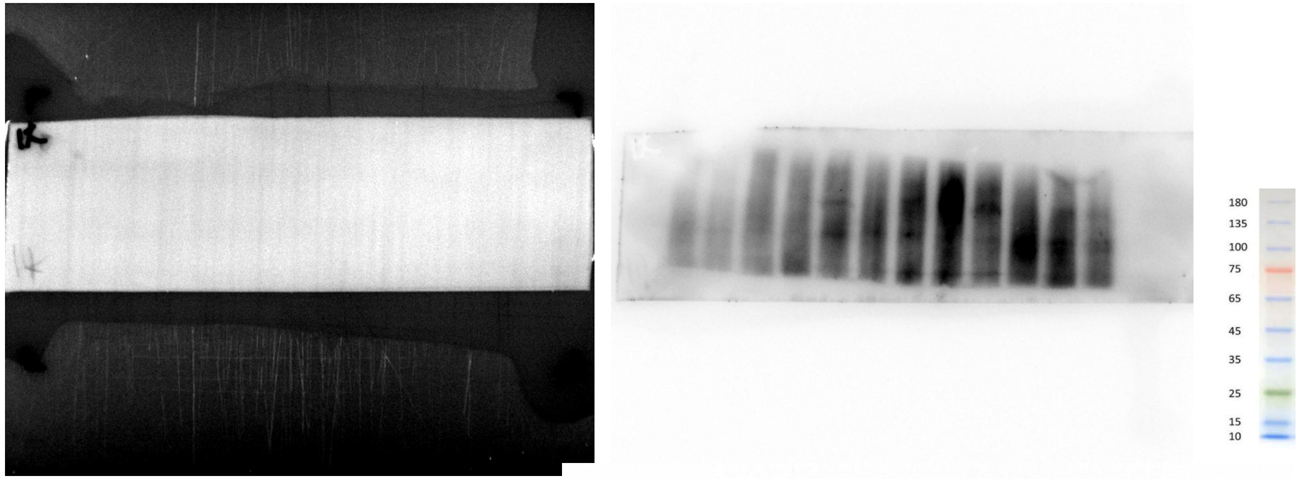

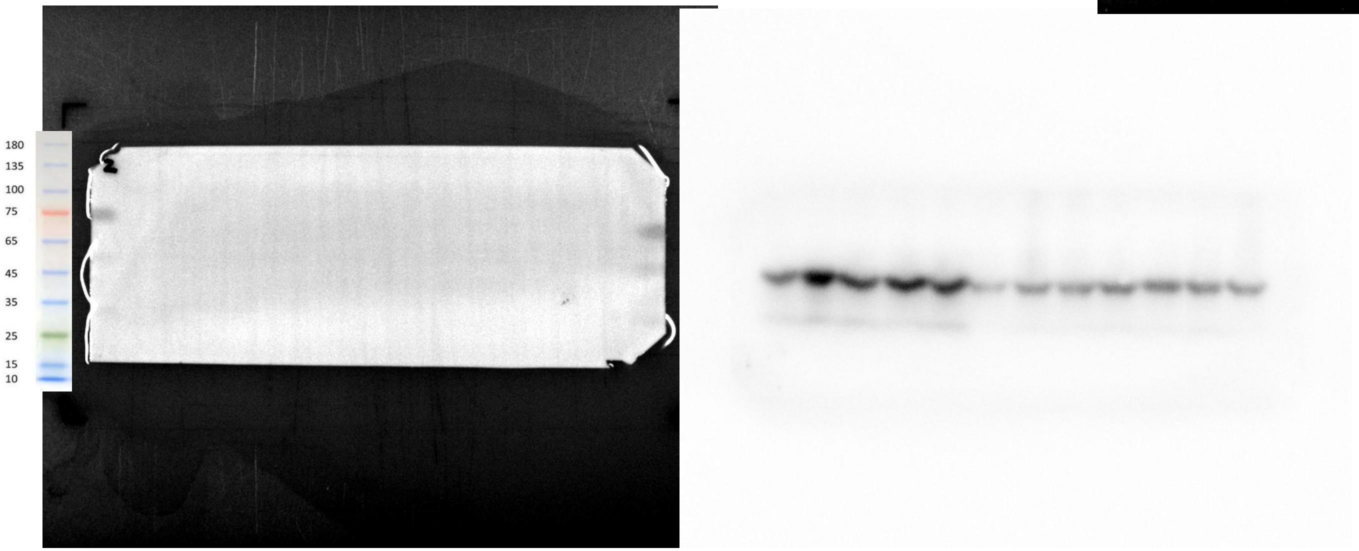

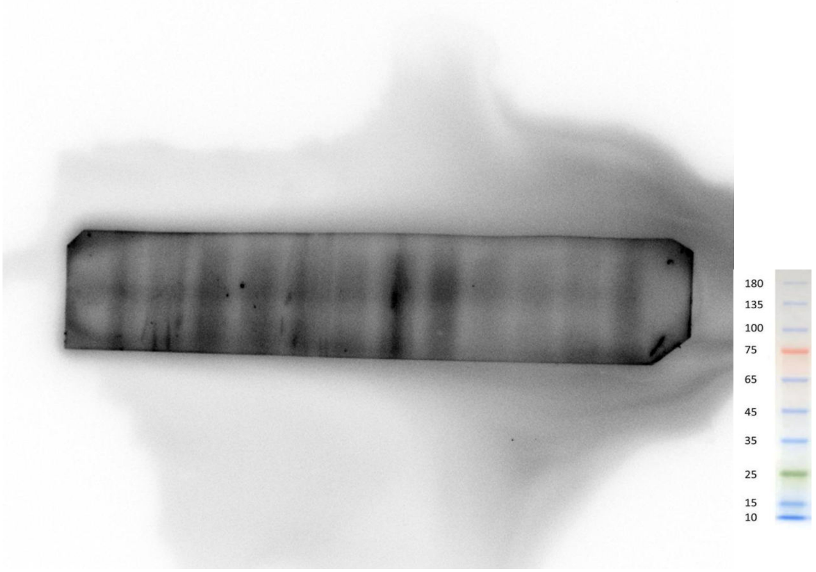

membrane cut at 75kDa, in the sequence of ZT2 control, ZT2 oua, ZT14 Control, ZT14 Oua

FigureS1 NAc (Week1) GAPDH
No.2 small and No.3 small (molecules)

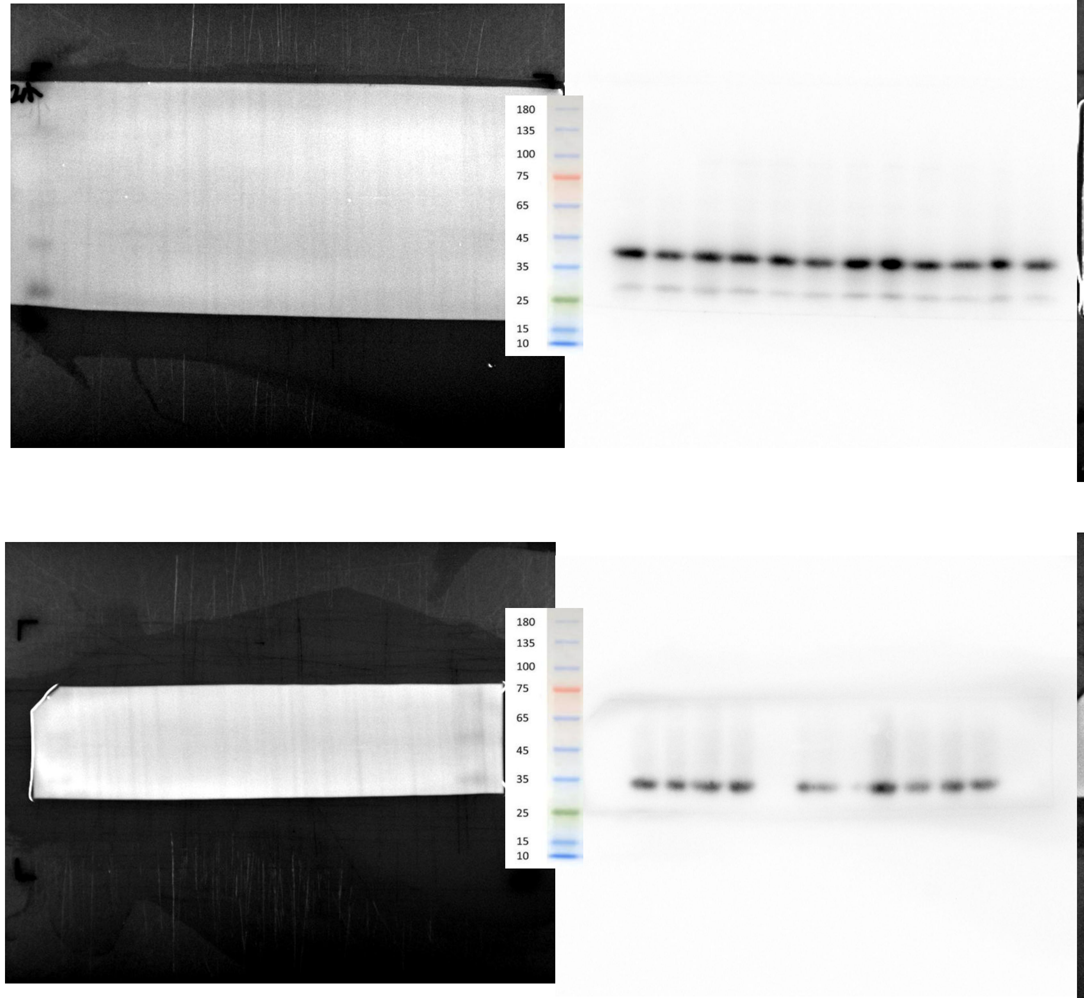

FigureS1 NAc (Week1) PER2
No.2 and 3 large (molecules)

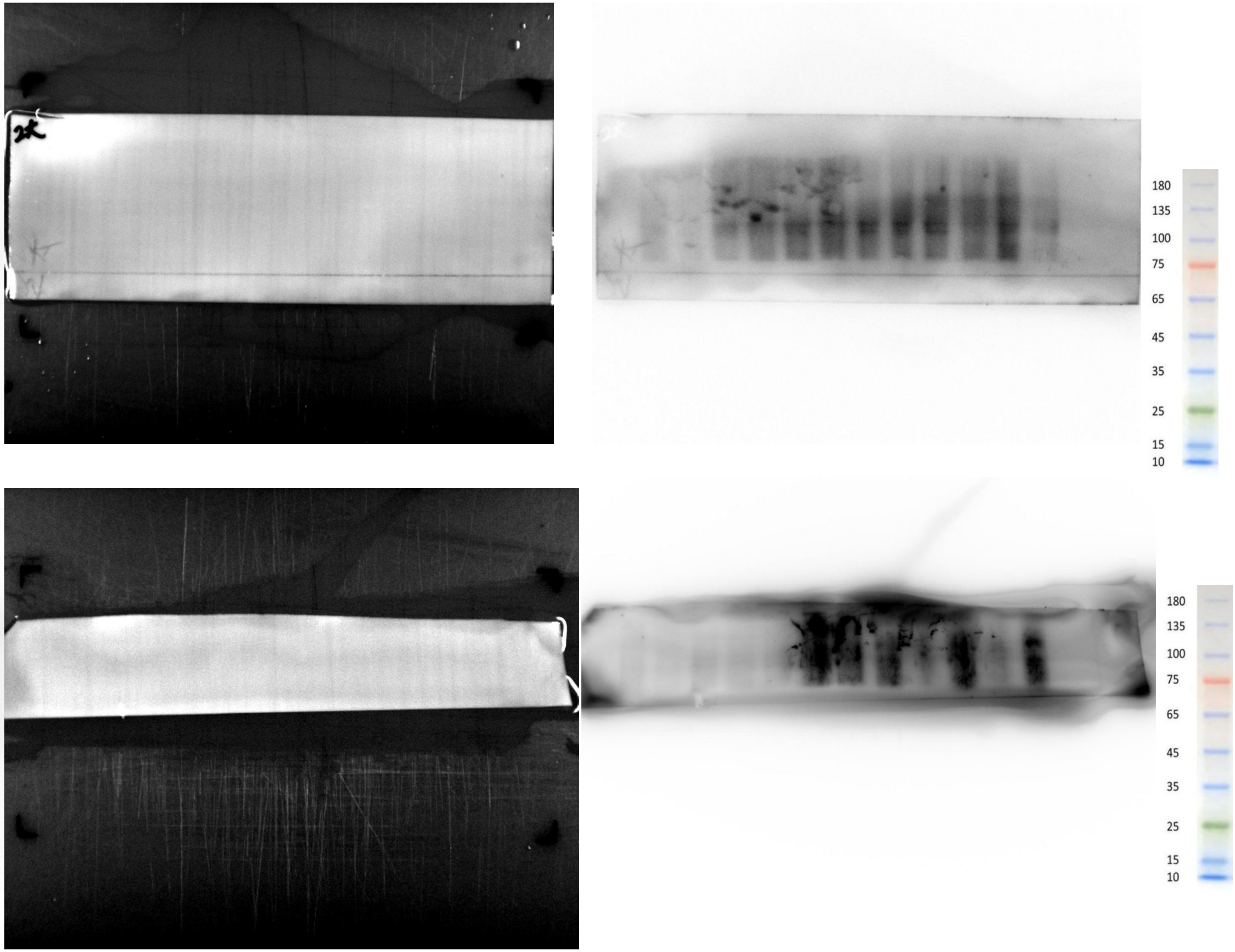

membrane cut at 75kDa, in the sequence of ZT2 control, ZT2 oua, ZT14 Control, ZT14 Oua

FigureS1 CA1 (Week2) GAPDH
No.1 small and No.2 small (molecules)

FigureS1 CA1 (Week2) PER2
No.1 and 2 large (molecules)

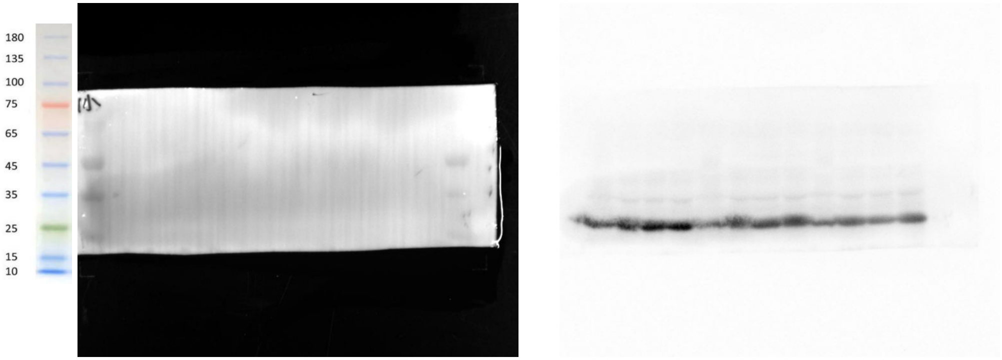

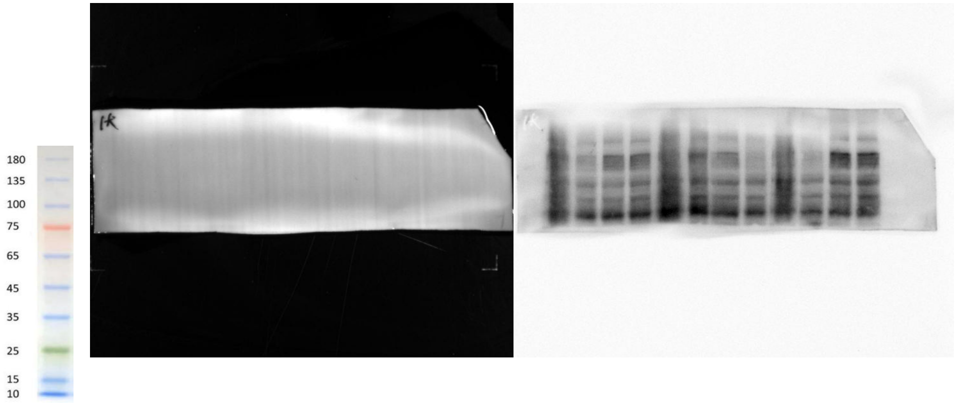

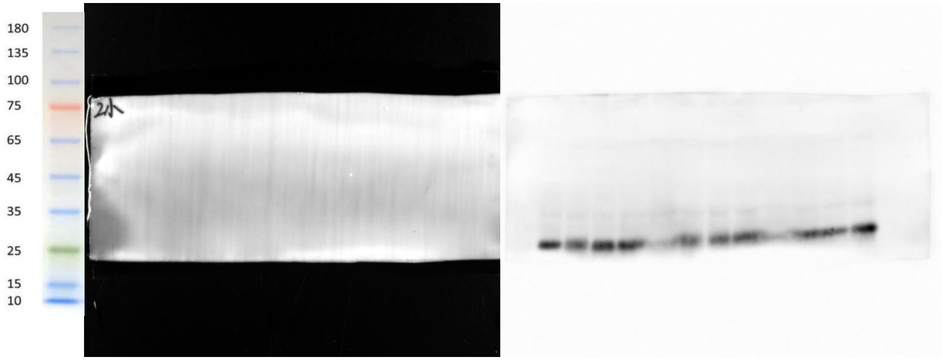

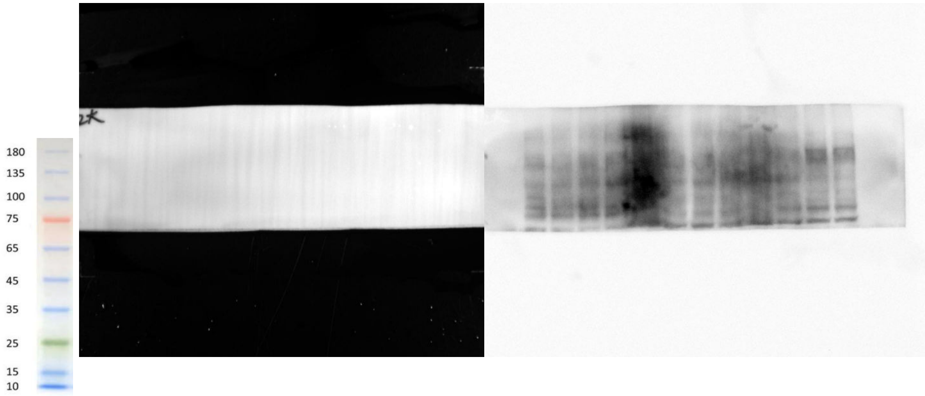

membrane cut at 75kDa, in the sequence of ZT2 control, ZT2 oua, ZT14 Control, ZT14 Oua

FigureS1 CA1 (Week2) pCREB
No.1 small and No.2 small (molecules)

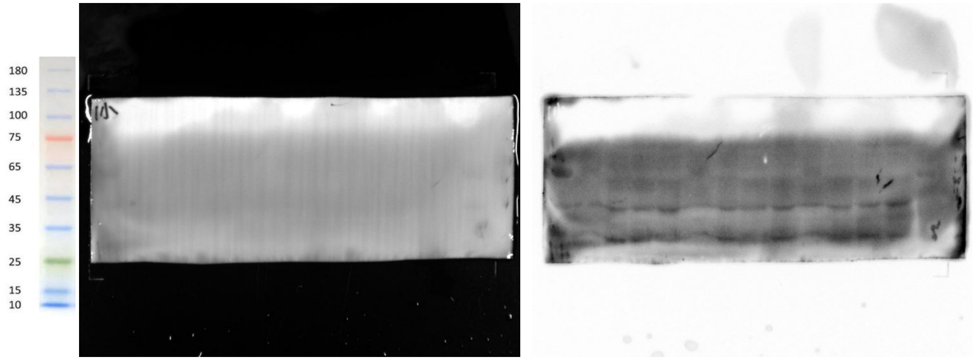

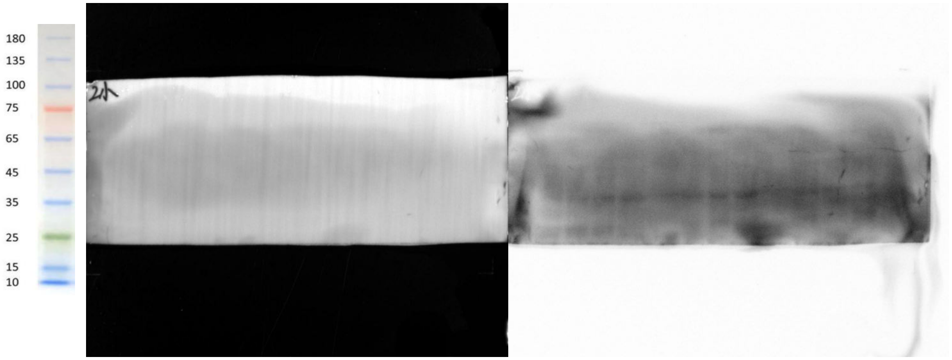

FigureS1 CA1 (Week2) PER1
No.1 and 2 large (molecules)

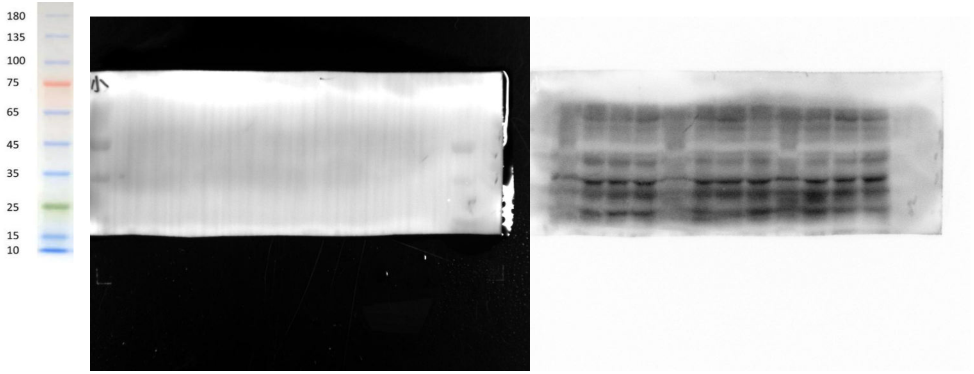

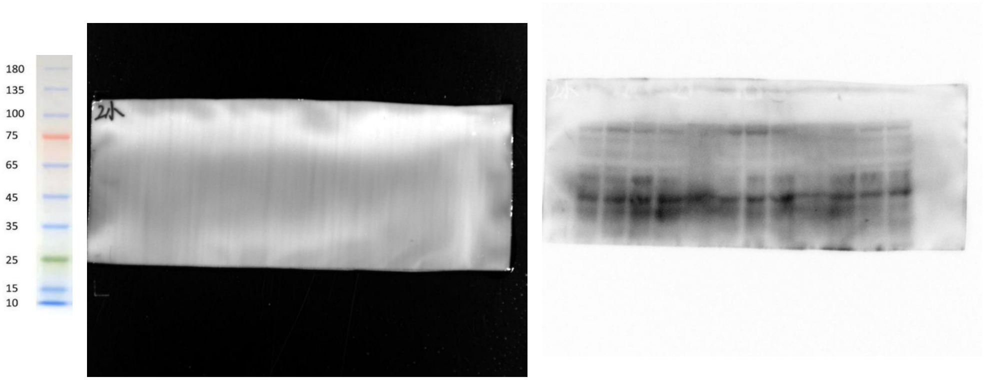

membrane cut at 75kDa, in the sequence of ZT2 control, ZT2 oua, ZT14 Control, ZT14 Oua

FigureS1 CA1 (Week2) CREB
No.3 small and No.4 small (molecules)

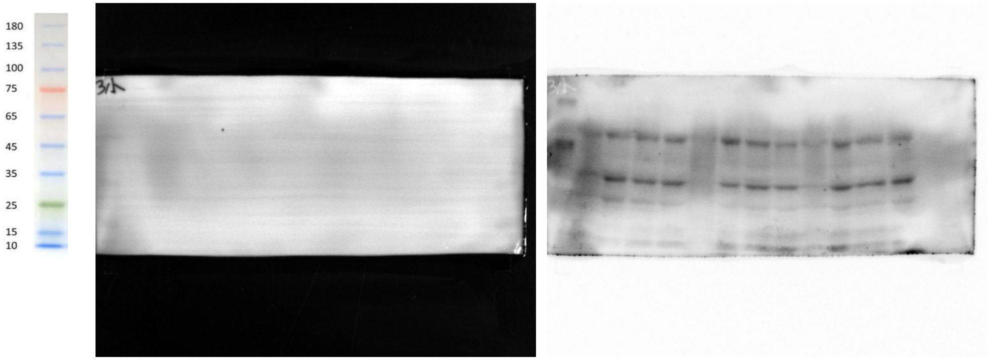

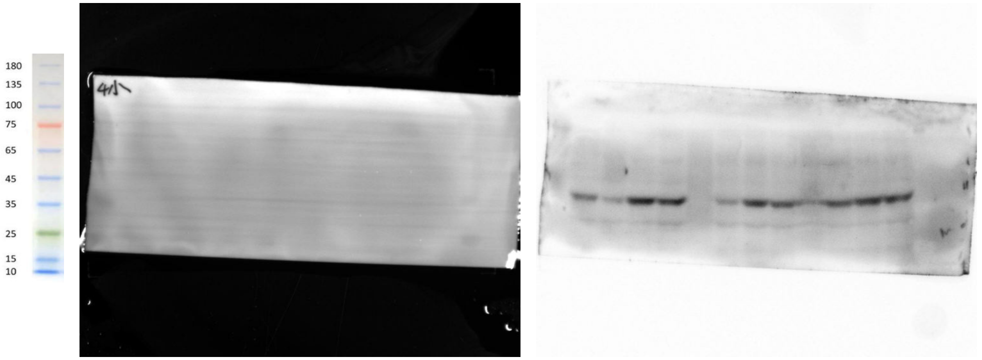

membrane cut at 75kDa, in the sequence of ZT2 control, ZT2 oua, ZT14 Control, ZT14 Oua

Supplementary Table S2. Detailed information about the utilized viral vectors

Full name	Short name	Target Seq	Target gene	Gene ID	Vector Name	Titer	Vector map	Blast result (below)
AAV9-pCAG-EGFP-pU6-shPer2/Scramble (PSC106389-1)	AAV-shPer2 AAV-Scramble	CCACACTTGCCTCTGAAATAA	Rat *Per2*	63840	GV478	4.31E+12 v.g./mL	Fig. S2A	Suppl. Result 1
AAV9-shCREB/AAV-Scramble (PSC110997-1)	AAV-shCREB AAV-Scramble	GCACTTAAGGACCTTTACTGC	Rat *Creb1*	81646	GV478	6.50E+12 v.g./mL	Fig. S4A	Suppl. Result 2
LV-pCMV-EGFP-Per2 (KL74162-1)	LV-Per2 LV-Control	GenBank ID: NM_031678.2	Rat *Per2*	63840	GV218	7.90E+08 TU/mL	Fig. S3A	Suppl. Result 3
LV-pCMV-EGFP-CREB (KL84917-1)	LV-CREB LV-Control	GenBank ID: NM_031017.2	*Rat Creb1*	81646	GV218	1.30E+08 TU/mL	Fig. S5A	Suppl. Result 4

Supplementary Result 1. AAV9-pCAG-EGFP-pU6-shPer2 blast result:

>PSC106389-1-pGCSIL-F_D05.ab1NNNNNGCNGTTAGAGAGNAATTGGATTAATTTGACTGTAAACACAAAGATATTAG
TACAAAATACGTGACGTAGAAAGTAATAATTTCTTGGGTAGTTTGCAGTTTTAAAATTATGTT
TTAAAATGGACTATCATATGCTTACCGTAACTTGAAAGTATTTCGATTTCTTGGCTTTATATA
TCTTGTGGAAAGGACGAAACACCGGCCACACTTGCCTCTGAAATAATTCAAGAGATTATTTC
AGAGGCAAGTGTGGTTTTTTAGAAATAGCAAGTTAAAATAAGGCTAGTCCGTTATCAACTTG
AAAAAGTGGCACCGAGTCGGTGCTTTTTTTCTGCAGCGGCTTCGGAATAGGAACTTCCCAGA
CATGATAAGATACATTGATGAGTTTGGACAAACCACAACTAGAATGCAGTGAAAAAAATGC
TTTATTTGTGAAATTTGTGATGCTATTGCTTTATTTGTAACCATTATAAGCTGCAATAAACAA
GTTGTTAACGTTAGGGGCGGGATAGCTAGAGCCAGACATGATAAGATACATTGATGAGTTTG
GACAAACCACAACTAGAATGCAGTGAAAAAAATGCTTTATTTGTGAAATTTGTGATGCTATT
GCTTTATTTGTAACCATTATAAGCTGCAATAAACAAGTTCCTCTCACTCTCTGATATTCATTT
CTTTGCAAGTTATAAATACTGAATAATAAGATGACATGAACTACTACTGCTAGAGATTTTCC
ACACTGACTAAAAGGGTCTGAGGGATCTCTAGTTACCAGAGTCACACAACAGACGGGCACA
CACTACTTGAAGCACTCAAGGCAAGCTTTATTGAGGCTTAAGCAGTGGGTTCCCTAGTTAGC
CAGAGAGCTCCCAGGCTCAGATCTGGTCTAACCAGAGAGACCCAGTACAGCAAAAAGCAGA
TCTTATCTTCGTTGGGAGTGAATTAGCCCTTCCAGTCCCCCCTTTTCTTTTAAAAGTGGCTAG
ATCTACAGCTGCCTTGTAGTCATGGTCTAAAGGTACCAGCCGGGAGCGGCCCAAAGGNGAT
CGACTCGTCTGAGGCGAAGCGAGACNCGNNAGCCGCAAGTCGCACAGCCGCCGNAAGNNGT
CCGCTGA

PSC106389-1 accggCCACACTTGCCTCTGAAATAAttcaagagaTTATTTCAGAGGCAAGTGTGGttttt

Supplementary Result 2. AAV9-shCREB/AAV-Scramble blast result:

>PSC110997-1-pGCSIL-F_E07.ab1NANTNNNNNGCTGTTAGAGAGTNATTGGAATTAATTTGACTGTAAACACAAAGAT
ATTAGTACAAAATACGTGACGTAGAAAGTAATAATTTCTTGGGTAGTTTGCAGTTTTAAAAT
TATGTTTTAAAATGGACTATCATATGCTTACCGTAACTTGAAAGTATTTCGATTTCTTGGCTT
TATATATCTTGTGGAAAGGACGAAACACCGGGCACTTAAGGACCTTTACTGCTTCAAGAGAG
CAGTAAAGGTCCTTAAGTGCTTTTTTAGAAATAGCAAGTTAAAATAAGGCTAGTCCGTTATC
AACTTGAAAAAGTGGCACCGAGTCGGTGCTTTTTTTCTGCAGCGGCTTCGGAATAGGAACTT
CCCAGACATGATAAGATACATTGATGAGTTTGGACAAACCACAACTAGAATGCAGTGAAAA
AAATGCTTTATTTGTGAAATTTGTGATGCTATTGCTTTATTTGTAACCATTATAAGCTGCAAT
AAACAAGTTGTTAACGTTAGGGGCGGGATAGCTAGAGCCAGACATGATAAGATACATTGAT
GAGTTTGGACAAACCACAACTAGAATGCAGTGAAAAAAATGCTTTATTTGTGAAATTTGTGA
TGCTATTGCTTTATTTGTAACCATTATAAGCTGCAATAAACAAGTTCCTCTCACTCTCTGATA
TTCATTTCTTTGCAAGTTATAAATACTGAATAATAAGATGACATGAACTACTACTGCTAGAG
ATTTTCCACACTGACTAAAAGGGTCTGAGGGATCTCTAGTTACCAGAGTCACACAACAGACG

GGCACACACTACTTGAAGCACTCAAGGCAAGCTTTATTGAGGCTTAAGCAGTGGGTTCCCTA
GTTAGCCAGAGAGCTCCCAGGCTCAGATCTGGTCTAACCAGAGAGACCCAGTACAAGCAAA
AAGCAGATCTTATCTTCGTTGGGAGTGAATTAGCCCTTCCAGTCCCCCCTTTTCTTTAAAAGT
GGNTAAGATCTACAGCTGCCTTGTAAGTCATTGGNCTAAAGGTACCAGGCGGGAGCGNCCA
AGGGAGATCGACTCGTCTGAAGNNANNNGACGCGANANNGCNNNCNNAGCAGNGCGNANA
NNCCGCTGNTTGAGGCCGAAGNN

PSC110997-1 accggGCACTTAAGGACCTTTACTGCttcaagagaGCAGTAAAGGTCCTTAAGTGCttttt

Supplementary Result 3. LV-pCMV-EGFP-Per2 blast result:

AATTCTGGCCGTTTTTGGCTTTTTGTTAGACGAAGCTTGGGCTGCAGGTCGACTCTAGAGGA
TCCCGCCACCATGAATGGATATGTGGACTTTTCCCCAAGTCCCACCAGCCCCACCCAAGAGCC
AGGGGAGCCTCAACCCACCCAGGCTGTGCTCCAGGAAGACGTGGACATGAGCAGCGGCTCC
AGCGGAAATGAAAACTGCTCCACGGGGCGGGACTCTCAGGGCAGTGACTGTGACGACAGTG
GAAAGGAGCTGCGGATGTTAGTGGAATCGTCCAACACTCACCCCAGCCCTGACGATACCTTC
AGACTCATGATGACAGAGGCGGAGCATAACCCCTCCACAAGCGGCTGCAGTAGTGAGCAGTC
TGCCAAAGCTGACGCACACAAAGAGCTGATAAGGACCCTGAGGGAGCTGAAGGTCCACCTC
CCTGCAGACAAGAAGGCCAAGGGGAAGGCCAGCACGCTGGCAACCTTGAAGTACGCTCTGC
GGAGCGTGAAGCAGGTGAAGGCTAATGAGGAGTACTACCAGCTGCTAATGTCCAGTGAGAGC
CAGCCCTGCAGCGTGGATGTGCCTTCCTACACCATGGAGCAGGTTGAGGGCATTACCTCCGAG
TATATTGTGAAGAACTCGGACATGTTTGCTGTGGCTGTGTCCCTGGTCTCTGGGAAGATCCTGT
ACATCTCCAACCAAGTCGCCCCCATCTTTCACTGTAAGAAGGACGCCTTCAGTGATGCCAAGT
TTGTGGAGTTCCTGGCTCCCCATGACGTCAGTGTGTTCCACAGCTACACCACCCCTTACAAGC
TTCCGCCCTGGAGTGTGAGCAGTGGCTTAGATTCTTTCACTCAGGAGTGCATGGAGGAGAAAT
CTTTTTTCTGCCGTGTCAGTGTTGGGAAACACCACGAGAATGAGATTCGCTACCAGCCCTTCC
GCATGACACCCTACCTGGTCAAGGTGCAAGAGCAGAAGGGCGCTGCGAGCCAGCTCTGCTGC
CTGCTGCTAGCAGAGAGGGTACACTCAGGCTATGAAGCTCCTAGAATTCCTCCCGAGAAGAG
AATTTTCACAACAACCCACACACCAAACTGCCTGTTCCAGGATGTGGACGAAAGGGCGGTCC
CCCTCCTGGGCTATCTACCTCAGGATCTGATCGAGACCCCTGTGCTCGTGCAGCTCCACCCCA
GCGACCGGCCCTTGATGCTCGCCATCCACAAGAAGATCCTACAGGCCAGTGGGCAGCCTTTC
GATTATTCTCCCATTCGATTCCGCACACGCAACGGGGAGTACATCACACTGGACACTAGCTGG
TCCAGCTTCATCAACCCGTGGAGCAGGAAGATATCCTTCATCATCGGGAGGCACAAAGTCAG
GGTAGGCCCTTTGAATGAGGATGTGTTCGCAGCCTCCCCTTGCCCAGAGGAGAAGACTCCGC
ACCCCAGCGTTCAGGAGCTCACAGAGCAAATCCACCGGCTACTGATGCAACCTGTCCCCCAC
AGCGGCTCCAGTGGCTATGGGAGCCTGGGCAGTAACGGATCCCACGAACACCTCATGAGCCA
GACATCATCCAGCGACAGCAATGGTCAAGAGGAGTCTCACTGGAGGAGATCCGGAATTTTTA
AAACCAGTGGCAAGAGTCAAAGCAAAAGTCACTTTTCTCCTGAGTCTGGAGGACAAAAGGA
AGCCTCTGTTGCAGAAATGCAAAGTAGTCCTCCAGCTCAGGTGAGGTCTGTCACCACCATGG
AAAGGGACAGCTCGGGGGCCAGCCTACCCAAGGCTAGCTTTCCAGAGGAACTAACCTATAAG
AGCCAGCCTCCTTGCTCCTACCAGCAGATCAGCTGCCTGGACAGTGTCATCAGGTACCTGGAG
AGCTGCAACGAGGCAGCCACCCTGAAAAGAAAGTGCGAGTTCCCAGCCAACATCCCATCCC
GGAAGGCCACGGTCAGCCCTGGGCTGCACTCTGGAGAGGCAGCGCGGTCCTCCAAGGTGAC
CAGCCACACGGAGGTCAGTGCTCACTTGAGCTCCTTGGCGTTGCCGGGCAAGGCCGAGAGT
GTGGTGTCCCTCACCAGCCAGTGCAGCTACAGCAGCACCATCGTGCACGTGGGCGACAAAAA
GCCACAGCCTGAACTAGAGACAGTAGAAGATGTGGCCAGTGGGCCTGAGTCCCAGGATGATG
CAGCTGGTGGCCTCAGCCAAGAAAAGGGGTCTCTGCAGAAGCTAGGCCTCACCAAGGAAGT
TCTGGCTGCACATACCCAGAGAGAGGAACAGGGCTTCCTGCAGAGGTTCAGGGAAGTGAGC
AGGCTCGGTGCCCTGCAGGCTCACTGCCAGAACTATCTCCAGGAGCGGTCCCGAGCCCCAGC
AAGTGATCGAGGACTAAGAAATGCTTCTGGAATAGAATCATCTTGGAAAAAAACTGGAAAGA
ACAGGAAACTGAAGTCCAAGCGTGTCAAGACTCGAGACTCTTCTGAGAGCACAGGGTCTGG
GGGACCCGTGTCCCACCGACCTCCCCTCGTGGGCCTGAATGCCACAGCCTGGTCGCCCTCTG
ACACATCCCAGTCTAGCTGTCCCTCTGCACCATTCCCTGCTCCAGTGCCAGCTTACCCACTACC
TGTGTTCCCGGCACCTGGAATAGTATCCACACCAGGGACGGTGGTGGCACCACCTGCAGCCG

CCCACACCGGCTTCACCATGCCTGTTGTGCCTATGGGCACCCAGCCTGAATTCGCAGTGCAGC
CCCTGCCGTTCGCTGCCCCCTTGGCTCCGGTCATGGCCTTCATGCTACCCAGCTACCCGTTTCC
ACCAGCAACCCCAAACCTGCCTCAGGCCTTCTTCCCCAGCCAGCCTCACTTTCCGGCCCATCC
CACACTTGCCTCTGAAATAACTCCTGCCTCCCAGGCTGAGTTCCCTAGTCGGACCTCGATGCT
CAGGCAGCCATGTGCTTGCCCAGTCACCCCCCCGGCTGGCACAGTGGCCTTGGGCAGAGCCT
CCCCGCCACTCTTCCAGTCCCGAGGCAGCAGTCCCCTACAGCTTAACCTGCTTCAGCTAGAGG
AAGCACCTGAAAGTAGTACTGGAGCTGCAGGGACCTTGGGGACCACGGGGACAGCAGCTTC
TGGTCTGGACTGCACATCTGGCGCATCTCGGGACCGGCAGCCAAAGGCACCTCCAACATGCA
GTGAGCCCTCAGACACCCAGAACAGTGATGCCATCTCCACCTCCAGTGACCTGCTCAACCTC
CTCCTGGGCGAGGACCTCTGCTCAGCCACCGGCTCAGCACTGTCGAGAAGCGGGGCATCTGC
CACCTCAGACTCACTGGGCTCCAGCTCCCTGGGCTGTGACACATCCCGGAGTGGGGCAGGCA
GCAGTGATACAAGTCACACCAGCAAATACTTTGGAAGCATTGACTCTTCAGAGAATAATCACA
AAGCAAAAATGATCACAGACACGGAGGAGAGTGAACAGTTCATTAAGTACGTCTTGCAGGAC
CCCATCTGGCTGCTGATGGCCAACACAGACGACAATATCATGATGACATACCAGCTGCCCTCC
CGGGATCTCCAGGCGGTCTTGAAAGAGGACCAGGAGAAGCTGAAGCTGCTGCAGAGGTCCC
AGCCCCACTTCACGGAGGGCCAGAGGCGAGAGCTTCGAGAGGTTCATCCGTGGGTCCACAC
CGGGGGTCTGCCTACCGCCATCGACGTAACAGGGTGTGTTTACTGTGAAAGTGAGGAGAAAG
GCAACCTTTGTCTGCCATATGAGGAAGACAGTCCTTCCCTGGGACTCTGTGATACCTCAGAAG
CCAAAGAGGAGGAGAGCGGACAGCTGGCCAATCCTAGGAAGGAGGCCCAGACGCCGGTCGC
CACCATGGTGAGCAAGGGCGAGGA

Supplementary Result 4. LV-pCMV-EGFP-CREB blast result:

TTCTGGCCGTTTTTGGCTTTTTTGTTAGACGAAGCTTGGGCTGCAGGTCGACTCTAGAGGATC
CCGCCACCATGACCATGGACTCTGGAGCAGACAACCAGCAGAGTGGAGATGCTGCTGTAACA
GAAGCTGAAAGTCAACAAATGACAGTTCAAGCCCAGCCACAGATTGCCACATTAGCCCAGGT
ATCCATGCCAGCAGCTCATGCGACGTCATCTGCTCCCACTGTAACCTTAGTGCAGCTGCCCAA
TGGGCAGACAGTCCAGGTCCATGGGGTCATCCAGGCGGCCCAGCCATCAGTTATTCAGTCTCC
ACAAGTCCAAACAGTTCAGTCTTCCTGTAAGGACTTAAAAAGACTTTTCTCCGGAACTCAGAT
TTCAACTATTGCAGAAAGTGAAGATTCACAGGAGTCTGTGGATAGTGTAACTGATTCCCAAAA
ACGAAGGGAAATCCTTTCAAGGAGGCCTTCCTACAGGAAAATTTTGAATGACTTATCTTCTGA
TGCACCAGGGGTGCCAAGGATTGAAGAAGAAAAATCAGAAGAAGAGACTTCAGCCCCTGCC
ATCACCACTGTAACAGTGCCAACCCCGATTTACCAAACTAGCAGTGGGCAGTATATTGCCATTA
CCCAGGGAGGAGCAATACAGCTGGCTAACAATGGTACCGATGGGGTACAGGGCCTGCAGACA
TTAACCATGACCAATGCAGCTGCCACTCAGCCGGGTACTACCATTCTACAATATGCACAGACC
ACTGATGGACAGCAGATTCTAGTGCCCAGCAACCAAGTTGTTGTTCAAGCTGCCTCTGGTGAT
GTACAAACATACCAGATTCGCACAGCACCCACTAGCACCATTGCCCCTGGAGTTGTTATGGCG
TCCTCCCCAGCACTTCCTACACAGCCTGCTGAAGAAGCAGCACGAAAGAGAGAGGTTCGTCT
AATGAAGAACAGGGAAGCAGCAAGAGAATGTCGTAGAAAGAAGAAAGAATATGTGAAATGT
TTAGAGAACAGAGTGGCAGTGCTTGAAAACCAAAACAAAACATTGATTGAGGAGCTAAAAG
CACTTAAGGACCTTTACTGCCACAAGTCAGATCCGGTCGCCACCATGGTGAGCAAGGGCGAG
